# Improving the conjugation of organic ligands enhances the antenna effect and promotes the luminescence and optical imaging of chiral mononuclear Eu(iii) complexes

**DOI:** 10.1039/d5sc09594h

**Published:** 2025-12-26

**Authors:** Ru-Yan Li, Meng-Juan Tang, Yun-Lan Li, Fan Yang, Hua-Hong Zou, Hai-Ling Wang, Zhong-Hong Zhu

**Affiliations:** a School of Chemistry and Chemical Engineering, Guangxi Key Laboratory of Electrochemical Energy Materials, Guangxi University Nanning 530004 P. R. China whling@gxu.edu.cn zzhong@gxu.edu.cn; b Key Laboratory for Chemistry and Molecular Engineering of Medicinal Resources (Ministry of Education of China), Guangxi Key Laboratory of Chemistry and Molecular Engineering of Medicinal Resources, School of Chemistry and Pharmaceutical Sciences, Guangxi Normal University Guilin 541004 P. R. China

## Abstract

Regulating the conjugated structure of organic ligands to enhance the molar absorption coefficient can effectively promote the energy transfer process of the antenna effect and is one of the most effective ways to construct lanthanide complex emitters with bright luminescence. Herein, 2-pyridinecarboxaldehyde was condensed with (1*R*,2*R*/1*S*,2*S*)-1,2-diphenylethylenediamine under *in situ* conditions to form ((1*E*,1′*E*)-*N*,*N*′-(1,2-diphenylethane-1,2-diyl)bis(1-(pyridin-2-yl)methanimine)) (L2), which was further coordinated with Eu(iii) ions to obtain a pair of dynamic chiral mononuclear Eu(iii) complex isomers (*R*/*S*-Eu) with aggregation-enhanced antenna effect behavior. Remarkably, in the single-molecule state, *R*/*S*-Eu exhibit no significant emission, while in the aggregated state, they both exhibit bright red luminescence. Density functional theory (DFT) calculations demonstrate that the energy levels of the ligand L2 closely match those of *R*-Eu, ensuring efficient ligand–metal energy transfer. The mononuclear Eu(iii) complex isomers *R*/*S*-Eu exhibited obvious circularly polarized luminescence (CPL) performance, with luminescence asymmetry factors (*g*_lum_) of 0.072/0.068, 0.159/0.073, and 0.045/0.002 at 511, 523, and 535 nm, respectively. Furthermore, *R*/*S*-Eu-DMSO demonstrated high-resolution optical imaging of various living cells and was specifically localized to lysosomal organelles. The chiral complex *R*-Eu-DMSO was primarily taken up by the zebrafish yolk sac and liver, exhibiting bright red luminescence and excellent optical imaging performance. This work not only provides a new method for improving the photophysical properties of chiral lanthanide complex isomers, but also opens up new horizons for expanding the bio-optical imaging applications of chiral lanthanide complex isomers.

## Abstract 2

*R*/*S*-Eu exhibited an aggregation-enhanced antenna effect in aqueous solution *via* a restricted intramolecular rotation mechanism, enabling high-resolution optical imaging of living cells and zebrafish.

## Graphical abstract

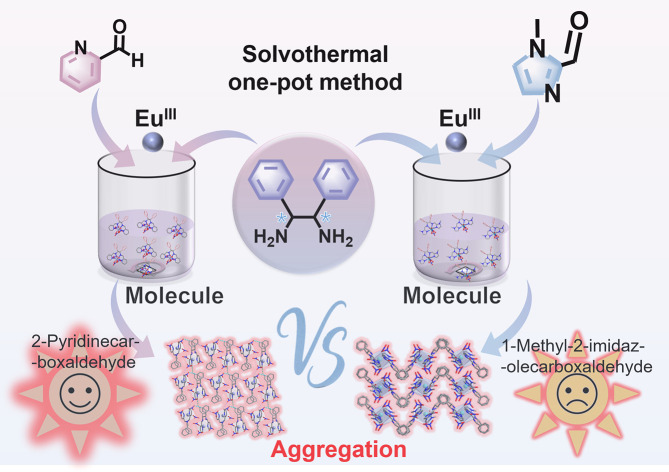

## 1 Introduction

Lanthanide ions have a complex 4f^n^ electron configuration, resulting in lanthanide complex emitters with numerous advantages, including high color purity, large Stokes shifts, and long excited-state lifetimes.^1–3^ These emitters have attracted widespread attention and have demonstrated significant advantages in solid-state lighting, bioimaging, smart sensing, high-security information storage, and other fields.^4–8^ The key to constructing lanthanide complexes with bright emission is to utilize the antenna effect to achieve efficient energy transfer.^9–11^ This process begins with the photoexcitation of an organic conjugated ligand: after the organic ligand absorbs energy, a π → π* transition occurs, and the electron is excited from the ground state to the lowest excited singlet state (S_1_), and then transferred to the lowest excited triplet state (T_1_) through intersystem crossing (ISC). When the T_1_ state energy level matches the energy gap (Δ*E*) of the excited state energy level of the lanthanide ions, the energy absorbed by the organic ligand will be effectively transferred to the lanthanide metal ions, ultimately realizing the characteristic luminescence of the lanthanide ions.^12,13^ Therefore, the light absorption ability of the ligand plays a decisive role in the antenna effect.^14–16^ Currently, the organic ligands that can match with lanthanide ions and produce antenna effects are mainly carboxylic acid ligands, such as iminodiacetic acid, salicylic acid, and benzoic acid.^17,18^ However, the complexes formed by the assembly of such ligands with lanthanide ions usually only exhibit static luminescence properties, making it difficult to achieve dynamic luminescence functions.^19–21^

In recent years, restriction of intramolecular motion (RIM) has been considered to be the most recognized theoretical model for constructing efficient and bright aggregation-induced emission fluorophores, which mainly includes the restriction of the intramolecular rotation (RIR) mechanism and the restriction of the intramolecular vibration (RIV) mechanism.^22–26^ Based on this, selecting organic ligands with obvious molecular rotor structures that can match the energy levels of lanthanide ions is an effective way to construct dynamic lanthanide complex emitters.^20^ In 2024, our team successfully prepared a dynamic chiral mononuclear Eu(iii) complex with aggregation-enhanced antenna effect behavior using chiral ligands containing molecular rotor units, and achieved a “double improvement” in the *g*_lum_ value (0.64) and *B*_CPL_ value (2429 M^−1^ cm^−1^).^19^ In addition, in the aggregated state, the close stacking of molecules in these lanthanide complexes containing molecular rotors can not only effectively shield the quenching effect of H_2_O molecules on the complexes, but also induce a significant luminescence enhancement effect. Next, we further realized the aggregation-enhanced antenna effect of dynamic chiral Eu(iii) complexes in aqueous solution for the first time through the RIR and RIV mechanisms, which significantly improved the red characteristic luminescence of the complexes in living cells and zebrafish systems, and successfully achieved high-resolution bio-optical imaging.^21^ Based on the above research, the key to optimizing the photophysical properties of lanthanide complexes lies in the precise design of the conjugated structure of the molecular rotor antenna.^27,28^ Compared with 1-methyl-1*H*-imidazole-2-carboxaldehyde, 2-pyridinecarboxaldehyde, with a higher degree of conjugation, has a more efficient energy transfer efficiency with metal ions and thus becomes an ideal choice for constructing high-performance dynamic luminescent lanthanide complexes.

**Scheme 1:**
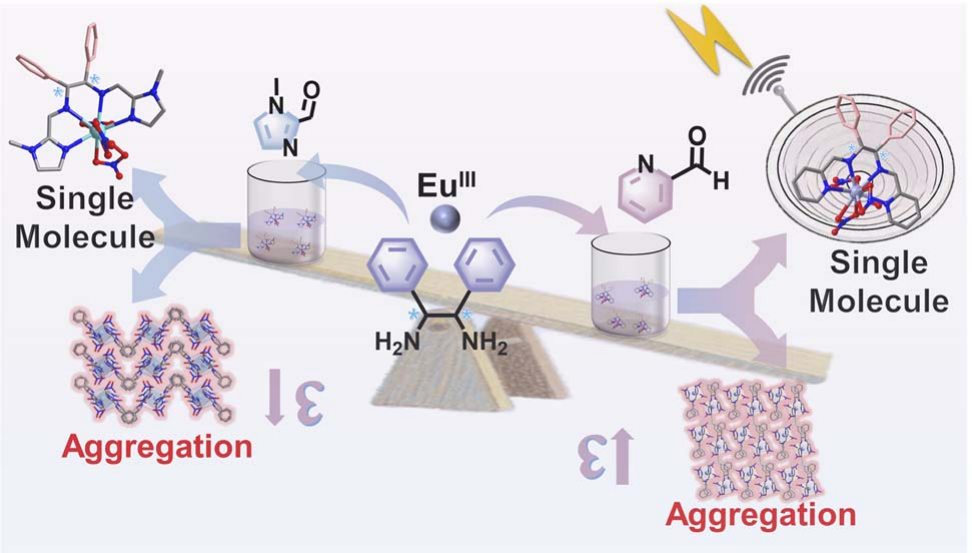
Schematic diagram of *R*-Eu with higher molar absorptivity constructed based on 2-pyridinecarboxaldehyde and 1-methyl-1*H*-imidazole-2-carboxaldehyde.

Herein, 2-pyridinecarboxaldehyde with a greater degree of conjugation to replace 1-methyl-1*H*-imidazole-2-carboxaldehyde, reacted with chiral (1*R*,2*R*/1*S*,2*S*)-1,2-diphenylethylenediamine under *in situ* conditions to generate the ligand ((1*E*,1′*E*)-*N*,*N*′-(1,2-diphenylethane-1,2-diyl)bis(1-(pyridin-2-yl)methanimine))(L2), which further reacted with Eu(iii) ions to obtain a pair of dynamic chiral Eu(iii) complex isomers *R*/*S*-Eu with AIE properties (Scheme 1). When Eu(iii) complexes aggregate in solution to form nanoparticles, the outer complex molecules form a protective layer by densely stacking, which not only effectively blocks the coordination attack of water molecules on the internal complexes but also significantly reduces the quenching effect of water molecule vibration on the characteristic emission of Eu(iii) ions. In the single-molecule state, the molecular rotors of *R*/*S*-Eu can rotate freely, while in the aggregated state, the rotation of the molecular rotors of *R*/*S*-Eu is restricted. With the increase in glycerol content, the emission intensity of *R*/*S*-Eu gradually increases. DFT calculations show that the HOMO energy level of ligand L2 (−5.3941 eV) is highly matched with the LUMO energy level of *R*-Eu (−5.4028 eV), providing favorable conditions for efficient ligand–metal energy transfer. Furthermore, *R*/*S*-Eu-DMSO not only achieve high-resolution cellular imaging of a variety of cells but also specifically localizes to lysosomal organelles. Furthermore, in zebrafish *in vivo* imaging studies, *R*-Eu-DMSO also demonstrated excellent high-resolution imaging performance, confirming its practical value as a biological imaging probe. This study not only expands the application prospects of dynamic chiral lanthanide complexes in the biomedical field but also provides new ideas for the design of chiral optical materials.

## 2 Results and discussion

### 2.1 Structural analysis of *R*/*S*-Eu

**Fig. 1:**
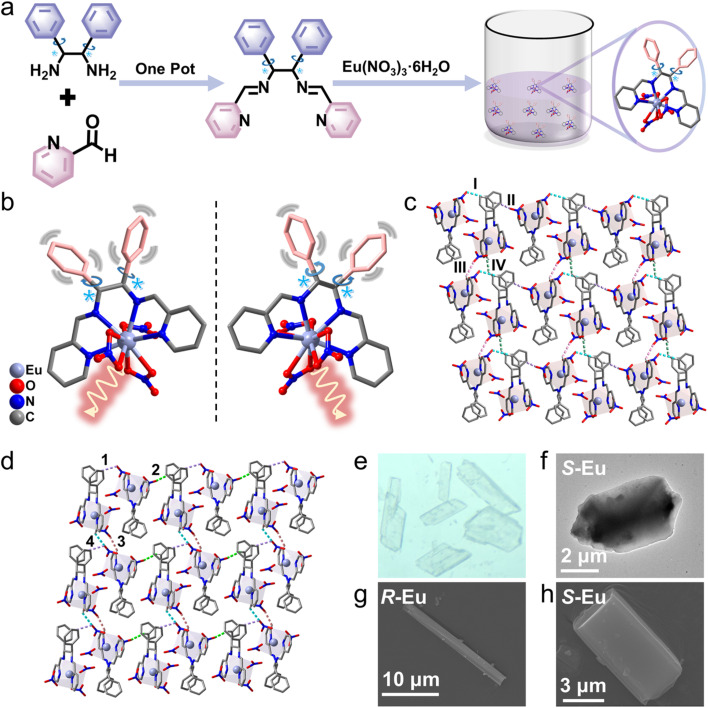
(a) Schematic diagram of the synthesis of *R*/*S*-Eu; (b) crystal structures of *R*-Eu (left) and *S*-Eu (right); (c and d) three-dimensional networks of *R*/*S*-Eu; (e) crystal image of *S*-Eu under daylight; (f) HR-TEM image of *S*-Eu; (g and h) SEM images of *R*/*S*-Eu.

Single crystal X-ray diffraction (SCXRD) reveals that colorless block crystals *R*/*S*-Eu crystals are in the triclinic *P*1 space group (Fig. 1a). *R*/*S*-Eu are composed of a Eu(iii) ion, a chiral Schiff base ligand L2, and three terminally coordinated NO_3_^−^ atoms. Its molecular formula is [Eu(*R*/*S*-L2)(NO_3_)_3_] (Fig. 1b). In the single-molecule state, the ligand L2 in the *R*/*S*-Eu structure has two freely rotating molecular rotors. In the aggregated state, the freely rotating molecular rotors in *R*/*S*-Eu and the nitrate groups in the adjacent molecules significantly restrict the free rotation of the benzene ring rotors through strong hydrogen bonds and steric hindrance. In addition, the independent units of *R*-Eu in the aggregated state are connected and stacked by four different C–H⋯O strong hydrogen bonds (I, C12–H12⋯O14 (2.469 Å); II, C17–H17⋯O17 (2.480 Å); III, C52–H52⋯O1 (2.479 Å); IV, C19–H19⋯O2 (2.510 Å)) to form a three-dimensional structure (Fig. 1c and S1a). The independent units of *S*-Eu in the aggregated state are also connected and stacked by four different C–H⋯O strong hydrogen bonds (1, C19–H19⋯O10 (2.507 Å); 2, C10–H10⋯O13 (2.475 Å); 3, C52–H52⋯O1 (2.446 Å); 4, C1–H1⋯O10 (2.454 Å)) to form a three-dimensional structure (Fig. 1d and S1b). Hydrogen bonds I and II/1 and 2 form a chain structure by connecting multiple independent units; the chains are connected by hydrogen bonds III and IV/3 and 4 to form a three-dimensional stacking structure. Structural analysis shows that the metal center Eu is in a coordination environment of *C*_2V_ sphenocorona J87 formed by N_4_O_6_ (Fig. S1c, d and Tables S5, 6). High-resolution transmission electron microscopy (HR-TEM) and scanning electron microscopy (SEM) results clearly show that *R*/*S*-Eu are block crystals with obvious regularity and a very clean surface (Fig. 1e–h). In addition, energy dispersive spectroscopy elemental mapping (EDS-mapping) shows that the three elements C, N, and O are evenly distributed in *S*-Eu (Fig. S2). The powder X-ray diffraction (PXRD) spectrum of polycrystalline *R*/*S*-Eu closely resembles the simulated spectrum, indicating that both are phase-pure (Fig. S3). The Fourier transform infrared (FT-IR) spectrum of *R*/*S*-Eu displays characteristic absorption peaks, which correspond to specific functional groups in the structure (Fig. S4). Thermal stability tests of *R*/*S*-Eu were carried out under a flowing nitrogen atmosphere at a rate of 5 °C min^−1^ slowly ramped up from 35 °C to 1000 °C. All the experimental results show that *R*/*S*-Eu have excellent thermal stability (Fig. S5).

### 2.2 Solid-state luminescence of *R*/*S*-Eu

**Fig. 2:**
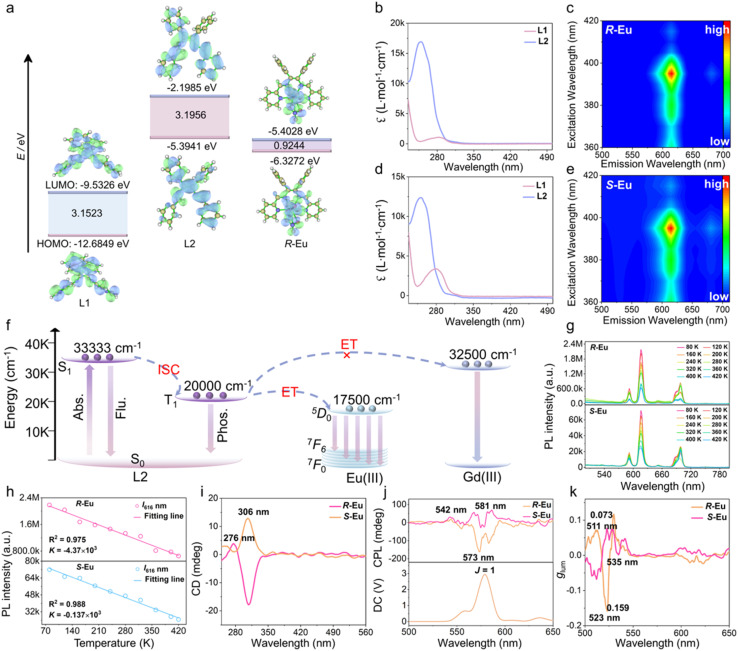
(a) Electron cloud density map of (1*E*,1′*E*)-*N*,*N*′-(1,2-diphenylethane-1,2-diyl)bis(1-(1-methyl-1*H*-imidazole-2-yl)methanimine) ligand (L1), (1*E*,1′*E*)-*N*,*N*′-(1,2-diphenylethane-1,2-diyl)bis(1-(pyridin-2-yl)methanimine) ligand (L2), and *R*-Eu obtained by DFT calculation; (b and d) UV-vis absorption spectra of ligands L1 and L2 (25 µM); (c and e) transient mode three-dimensional photoluminescence spectra of solid-state *R*/*S*-Eu; (f) Jablonski level diagram of *R*/*S*-Eu; (g) solid-state luminescence spectra of *R*/*S*-Eu at different temperatures; (h) relationship between the emission intensity at 616 nm and temperature of *R*/*S*-Eu at 80–420 K; (i) CD spectra, (j) CPL spectra, DC spectra, and (k) *g*_lum_ value of *R*/*S*-Eu.

Lanthanide complexes exhibit exceptional optical properties due to the unique configuration of their 4f^n^ electron shell. Notably, their emission spectra exhibit high monochromaticity, as the transition of 4f electrons to higher energy levels is minimally affected by the ligand field effect. These properties have led to the increasing potential of lanthanide complex-based luminescent materials in biomedical testing, solid-state light-emitting devices, and chemical sensing.^29–32^ In previous studies, we used 1-methyl-1*H*-imidazole-2-carboxaldehyde, (1*R*/*S*,2*R*/*S*)-(−/+)-1,2-diphenylethylenediamine, and Eu(NO_3_)_3_·6H_2_O as reaction raw materials and successfully obtained two examples of light yellow block crystals *R/S*-Me-Eu through solvothermal reactions. It is worth noting that the organic ligands used in the *R/S*-Me-Eu and *R*/*S*-Eu structures have different conjugation properties. To further explore the structure–activity relationship between the degree of ligand conjugation and the energy level matching of Ln(III) ions, a systematic computational study was carried out using DFT. Specifically, the GGA-PBE functional combined with the DND basis set was used to theoretically calculate the frontier orbital energy levels of ligands L1 and L2 and the *R*-Eu complex (including the highest occupied molecular orbital (HOMO) and the lowest unoccupied molecular orbital (LUMO)) (Fig. 2a). As shown in Fig. 2a, the HOMO and LUMO energy levels of ligand L1 are −12.6849 and −9.5326 eV, respectively, with a corresponding energy gap of 3.1523 eV; while the HOMO and LUMO energy levels of ligand L2 are −5.3941 and −2.1985 eV, respectively, with an energy gap of 3.1956 eV. The HOMO energy of *R*-Eu is −6.3272 eV, the LUMO energy is −5.4028 eV, and the energy gap is 0.9244 eV. It is worth noting that although the energy gaps of the two are similar, the degree of conjugation of ligand L2 is higher, and its HOMO energy level almost completely matches the LUMO energy level of *R*-Eu; this energy level alignment significantly promotes the energy transfer efficiency. Further UV-vis absorption spectroscopy tests confirmed that under the same test conditions, the molar absorption coefficient of ligand L2 was significantly higher than that of L1 (Fig. 2b and d). The above results together prove that ligand L2 is more conducive to achieving an effective antenna effect and energy transfer.

Under 365 nm ultraviolet light, it was observed that the *R*/*S*-Eu solid had bright red luminescence. To further explore the luminescence behavior of *R*/*S*-Eu in the aggregated state, their solid-state luminescence spectra were tested. When *R*/*S*-Eu were excited with 395 nm light, they all showed characteristic emission peaks at 590, 615, 648, and 682 nm, which were attributed to the ^5^*D*_0_ → ^7^*F*_1_, ^5^*D*_0_ → ^7^*F*_2_, ^5^*D*_0_ → ^7^*F*_3_, and ^5^*D*_0_ → ^7^*F*_4_ energy level transitions of Eu(iii) ions, respectively (Fig. S6a and b). The solid-state luminescence lifetimes of the *R*/*S*-Eu were also measured, showing long lifetimes of 1109.7 and 1045.6 µs (Fig. S7). In addition, the emission spectra of *R*/*S*-Eu dissolved in DMSO also show the characteristic emission peak of Eu(iii) ions, and their solution-state fluorescence absolute quantum yields (QYs) are 29.90% and 35.95%, respectively (Fig. S6c, d and S8). In the excitation range of 360–420 nm, the transient three-dimensional luminescence spectra of both solid *R*/*S*-Eu and liquid *R*/*S*-Eu-DMSO show a characteristic emission peak. Notably, the position of this emission peak does not shift significantly with changes in the excitation wavelength, indicating that the luminescence behavior of *R*/*S*-Eu and *R*/*S*-Eu-DMSO are not dependent on the excitation wavelength (Fig. 2c, e, S9a and 9b).

To further explore the energy transfer pathway and luminescence mechanism of *R*/*S*-Eu, a Jablonski energy diagram was drawn (Fig. 2f). According to Reinhoudt's rule of thumb, the intersystem crossing (ISC) process can only be effectively carried out when the energy difference between the excited singlet and triplet states of the ligand is greater than 5000 cm^−1^.^33,34^ The energy of the excited singlet state S_1_ of ligand L2 was calculated to be 33 333 cm^−1^ based on its solid-state UV-diffuse reflectance absorption spectrum. The lowest excited state energy level of the Gd(iii) ions (32 500 cm^−1^) is significantly higher than that of the singlet excited state of ligand L2, preventing energy transfer from the ligand to the Gd(iii) ions. Therefore, the phosphorescence spectrum of *R*-Gd was measured at 77 K. Gaussian fitting revealed that the energy of the triplet excited state T_1_ of ligand L2 was 20 000 cm^−1^, and the energy gap (Δ*E*) between the S_1_ and T_1_ states of L2 was 13 333 cm^−1^. This conforms to Reinhoudt's empirical rule, indicating that ligand L2 can effectively undergo the ISC process. The energy gap (Δ*E*) between the ligand's triplet excited state and the lowest excited state of Ln(III) ions must be within an appropriate range for efficient energy transfer. Latva's rule of thumb indicates that the optimal Δ*E* for energy transfer from Eu(iii) ions is 2000–5000 cm^−1^.^35–37^ Given that the energy value of the ^5^*D*_0_ level of the Eu(iii) ions is 17 500 cm^−1^, the calculated Δ*E* between the T_1_ state of the ligand L2 and the Eu(iii) ions is 2500 cm^−1^. The ligand L2 can effectively sensitize the luminescence of the Eu(iii) ions. Obviously, increasing the conjugation degree of the ligand can increase the absorbance and thus enhance the antenna effect, but the higher the conjugation degree of the ligand, the better. Only when the energy level difference between the T_1_ state of the ligand and the metal ion is within the optimal matching range, it is effective to regulate the conjugation degree of the ligand.

In addition, to explore the photophysical behavior of the molecular rotor in the structure in response to temperature, we obtained the temperature-dependent fluorescence spectra of *R*/*S*-Eu (Fig. 2g). The experimental results are shown in Fig. 2g; as the temperature increases from 80 K to 420 K, the characteristic emission intensity of Eu(iii) ions of *R*-Eu and *S*-Eu gradually decreases, and the luminescence intensity shows a good linear dependence on temperature, exhibiting obvious linear thermal quenching luminescence behavior (Fig. 2h). This linear thermal quenching phenomenon of luminescence has gradually been discovered in lanthanide complex emitters.^38,39^ This phenomenon is usually caused by the increase in temperature, which leads to the intensification of intramolecular vibration, thereby enhancing the non-radiative transition process of the ligand triplet energy level to the excited state of the lanthanide ions, and exciting the vibration energy level of the lanthanide ions itself, dissipating energy through the multi-phonon relaxation process.^40,41^ Both *R*/*S*-Eu exhibit obvious linear thermal quenching luminescence behavior, which can be attributed to the fact that the increase in temperature leads to the intensification of molecular rotor vibration, thereby significantly enhancing the non-radiative transition process.

### 2.3 Circularly polarized luminescence properties of *R*/*S*-Eu

Circularly polarized luminescent (CPL) materials have shown significant advantages in potential applications such as 3D display, information anti-counterfeiting, and biosensing due to their unique polarization form.^42–45^ Circular dichroism (CD) and luminescence are two key factors in the construction of circularly polarized luminescent (CPL) materials. The quantitative evaluation of CPL performance usually adopts two key parameters: the luminescence asymmetry factor (*g*_lum_) and CPL brightness (*B*_CPL_), where *g*_lum_ (*g*_lum_ = 2(*I*_L_ − *I*_R_)/(*I*_L_ + *I*_R_), *I*_L_ and *I*_R_ are the intensities of the emitted left and right circularly polarized light, respectively) reflects the intensity difference between left and right CPL.^46,47^ The unique magnetic dipole-allowed f–f transition characteristics of Ln(III) ions make them an ideal choice for constructing high-performance CPL materials, which usually exhibit a large *g*_lum_. Based on the chiral characteristics of the ligand, we verified the chirality of *R*/*S*-Eu through CD spectroscopy. As shown in Fig. 2i, it can be observed that both *R*/*S*-Eu exhibit well-matched mirror-image CD curves in the aggregated state, indicating that *R*-Eu and *S*-Eu are enantiomers of each other. The CD spectrum of *S*-Eu exhibits a positive Cotton effect at 276 and 306 nm, respectively, while *R*-Eu displays opposite signals at the same positions, forming a mirror image (Fig. 2i). The absorption peak at 276 nm can be attributed to the π → π* energy level transition of the pyridine ring in the ligand, and the absorption peak at 306 nm can be attributed to the n → π* energy level transition of –C=N–. Based on the CD and PL characteristics of *R*/*S*-Eu, we further tested the CPL performance of the above-mentioned complexes. Specifically, obvious mirror CPL signal peaks were observed for *R*/*S*-Eu at 542, 573, and 581 nm, respectively. The signal at 581 nm was attributed to the ^5^*D*_0_ → ^7^*F*_1_ energy level transition of the Eu(iii) ions (Fig. 2j). Generally, the *g*_lum_ values of chiral organic fluorophores, chiral transition metal complexes, and chiral inorganic materials are generally less than 10^−3^.^48–50^ Lanthanide ions usually exhibit high *g*_lum_ because they may have magnetic dipole-allowed transitions, especially Eu(iii) complexes, whose ^5^*D*_0_ → ^7^*F*_1_ energy level has high g_lum_ and has attracted widespread attention (Table S2). The corresponding |*g*_lum_| values of *R*/*S*-Eu at 511, 523, and 535 nm were 0.072/0.068, 0.159/0.073, and 0.045/0.002, respectively (Fig. 2k). The significant differences observed in the signals of the *R*-Eu and *S*-Eu enantiomers in the solid-state CPL spectra are mainly attributed to the inherent anisotropy of the solid sample. To mitigate the influence of anisotropy on CPL, the CPL performance of *R*/*S*-Eu dispersed in CHCl_3_ was further tested. Distinct mirror-symmetrical CPL signal peaks of *R*/*S*-Eu were observed at 531, 542, 560, and 581 nm, respectively (Fig. S9c). The corresponding |*g*_lum_| values for *R*/*S*-Eu at 514, 531, and 551 nm were 0.027/0.011, 0.011/0.042, and 0.078/0.031, respectively (Fig. S9d). All in all, *R*/*S*-Eu has a large *g*_lum_, indicating that it has good CPL performance.

### 2.4 Aggregation-induced emission characteristics of *R*/*S*-Eu

**Fig. 3:**
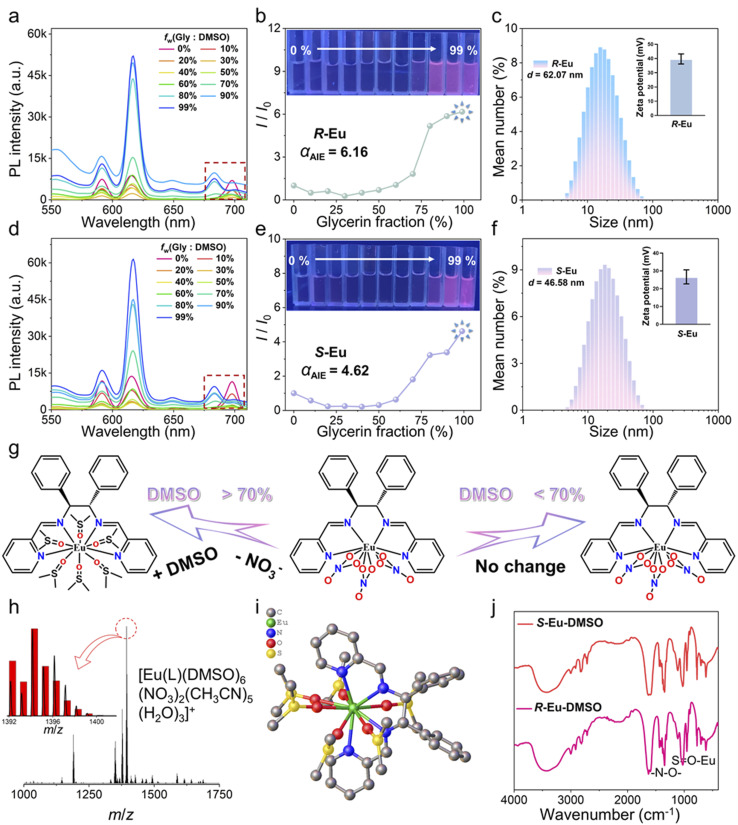
(a and d) Emission spectra of *R*/*S*-Eu in different Gly/DMSO mixed solutions under 395 nm excitation; (b and e) luminescence intensity of *R*/*S*-Eu at 617 nm as a function of *f*_w_. The inset shows the luminescence photos of *R*/*S*-Eu in Gly/DMSO mixed solutions with different ratios under UV light; (c and f) DLS results and zeta potential of *R*/*S*-Eu; (g) schematic diagram of the transformation between *R*/*S*-Eu and *R*/*S*-Eu-DMSO; (h) HRESI-MS spectrum of *S*-Eu-DMSO in positive ion mode; the inset shows a comparison of the experimental value (black) and simulated value (red) of the molecular ion peak at *m*/*z* = 1394.15; (i) optimized structure of *R*/*S*-Eu-DMSO by DFT calculation; (j) FT-IR absorption spectrum of *R*/*S*-Eu-DMSO.

Aggregation-induced emission (AIE) not only overcomes the bottleneck problem of aggregation-induced quenching of traditional fluorescent molecules, but also its materials show significant advantages in fields such as light-emitting devices and bioimaging.^51,52^ At present, the AIE system constructed by the mechanism of restricted intramolecular motion (such as restricted rotation/vibration) exhibits extremely high luminescence efficiency. The ingenious combination of organic ligands with molecular rotors or vibration units and lanthanide ions can not only effectively inhibit non-radiative transitions, but also greatly enhance the luminescence of metal central ions through ligand sensitization.^53–56^ In recent years, a variety of AIEgens and lanthanide complexes with AIE properties have been designed and synthesized.^57–59^ Based on the structural characteristics of *R*/*S*-Eu, we further studied their AIE properties. Specifically, *R*/*S*-Eu dissolved in DMSO exhibited no obvious luminescence, while the solution in glycerol exhibited red luminescence. Therefore, DMSO was selected as a good solvent and glycerol as a poor solvent. Equal amounts of *R*/*S*-Eu were dissolved in Gly/DMSO mixed solutions with different glycerol contents (*f*_w_), and their emission spectra were measured. The experimental results showed that both *R*/*S*-Eu exhibited luminescence quenching in the good solvent pure DMSO, and the luminescence intensity of the solution gradually decreased with increasing good solvent (DMSO) content. However, with increasing poor solvent (glycerol) content, the luminescence intensity of the solution gradually increased, indicating that both *R*/*S*-Eu exhibited typical AIE characteristics (Fig. 3a and d). The values of *α*_AIE_ calculated by using *I*/*I*_0_ and *R*/*S*-Eu were 6.16 and 4.62, respectively (Fig. 3b and e). The emission spectra of *R*/*S*-Eu showed that both exhibited characteristic emission peaks at 591, 616, 649, and 683 nm, which can be attributed to the ^5^*D*_0_ → ^7^*F*_1_, ^5^*D*_0_ → ^7^*F*_2_, ^5^*D*_0_ → ^7^*F*_3_, and ^5^*D*_0_ → ^7^*F*_4_ energy level transitions of Eu(iii) ions.^60^ It is worth noting that when the content of good solvent DMSO is >70%, the emission wavelength of the ^5^*D*_0_ → ^7^*F*_4_ energy level of Eu(iii) ions in *R*/*S*-Eu shifts significantly. At the same time, the UV-vis absorption spectrum of *R*/*S*-Eu in the mixed solution with a glycerol content of 99% did not start from the origin but showed a certain absorption value, indicating that *R*/*S*-Eu formed an aggregate suspension in the poor solvent glycerol (Fig. S10). We selected weakly coordinating solvents MeOH and DMF as good solvents to conduct comparative measurements of AIE titration curves. The luminescence spectra (especially the ^5^*D*_0_ → ^7^*F*_1_ energy level and the ^5^*D*_0_ → ^7^*F*_2_ energy level) were highly consistent with those in DMSO. It is noteworthy that when MeOH is a good solvent, the ^5^*D*_0_ → ^7^*F*_4_ energy level of the Eu(iii) ions does not shift; when DMF is a good solvent, the ^5^*D*_0_ → ^7^*F*_4_ energy level of the Eu(iii) ions shifts only when the DMF content is greater than 80% (Fig. S11). *R*/*S*-Eu were dispersed in a mixed solvent (*V*_DMSO_/*V*_water_ = 1/99), and the particle sizes of *R*/*S*-Eu were measured by dynamic light scattering to be 62.07 and 46.58 nm, respectively. In addition, the zeta potentials of *R*/*S*-Eu in the aggregated state were 39.7 and 26.67 mV, respectively, indicating that their aggregates can remain stable (Fig. 3c and f).

In Gly/DMSO mixed solutions with DMSO content higher than 70%, the emission wavelength of the ^5^*D*_0_ → ^7^*F*_4_ energy level of the Eu(iii) ions of the chiral isomer *R*/*S*-Eu shifted significantly (Fig. 3g). High-resolution electrospray ionization mass spectrometry (HRESI-MS) was further used to deeply explore the solution-state behavior of *R*/*S*-Eu dissolved in DMSO (Fig. 3h and S12–S15). It is worth noting that the molecular ion peaks of HRESI-MS of *R*/*S*-Eu dissolved in DMSO are highly consistent with the main framework of *R*/*S*-Eu-DMSO in which the terminal-coordinated NO_3_^−^ ions are completely replaced by DMSO. The above molecular ion peaks can be attributed to [Eu(L)(DMSO)_6_(NO_3_)(OH)(CH_3_OH)_5_(H_2_O)_8_]^+^ (exp. 1394.15; cal. 1394.39) and [Eu(L)(DMSO)_6_(NO_3_)_2_(CH_3_CN)_5_(H_2_O)_3_]^+^ (exp. 1394.15; cal. 1394.32). Obviously, the above results prove that the terminal-coordinated NO_3_^−^ ions of the chiral isomer *R*/*S*-Eu dissolved in DMSO are replaced by six DMSO molecules to form *R*/*S*-Eu-DMSO. In addition, density functional theory (DFT) calculations were further used to optimize the structure of *R*/*S*-Eu-DMSO, fully considering factors such as the electronic structure and geometric configuration of the system. The optimized structural model obtained in the end clearly showed that the six DMSO molecules effectively coordinated with the Eu(iii) ions through the lone pairs of electrons on their oxygen atoms to form *R*/*S*-Eu-DMSO, which was consistent with the results of the molecular ion peak of HRESI-MS (Fig. 3i). Finally, the FT-IR absorption spectroscopy results of *R*/*S*-Eu-DMSO also proved that the guest molecule DMSO replaced the terminal-coordinated NO_3_^−^ ions, thereby coordinating with the Eu(iii) ions (Fig. 3j).

### 2.5 Biological imaging of *R*/*S*-Eu-DMSO

**Fig. 4:**
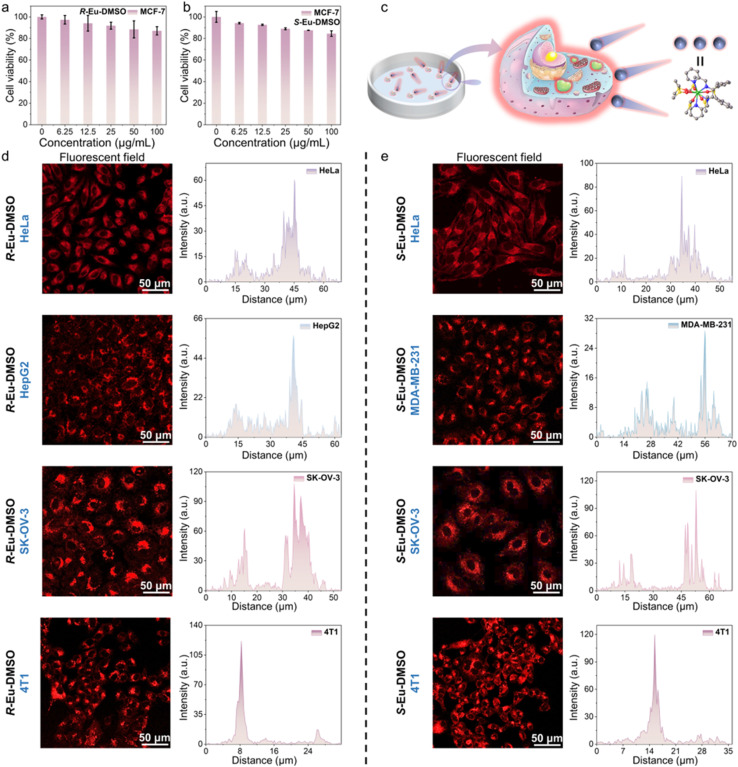
(a and b) Cell viability after co-incubation of MCF-7 cells with different concentrations of *R*/*S*-Eu-DMSO; (c) schematic diagram of cell imaging; (d) CLSM images and quantitative analysis of fluorescence intensity after co-incubation of HeLa, HepG2, SK-OV-3, and 4T1 cells with *R*-Eu-DMSO; (e) CLSM images and quantitative analysis of fluorescence intensity after co-incubation of HeLa, MDA-MB-231, SK-OV-3, and 4T1 cells with *S*-Eu-DMSO.

**Fig. 5:**
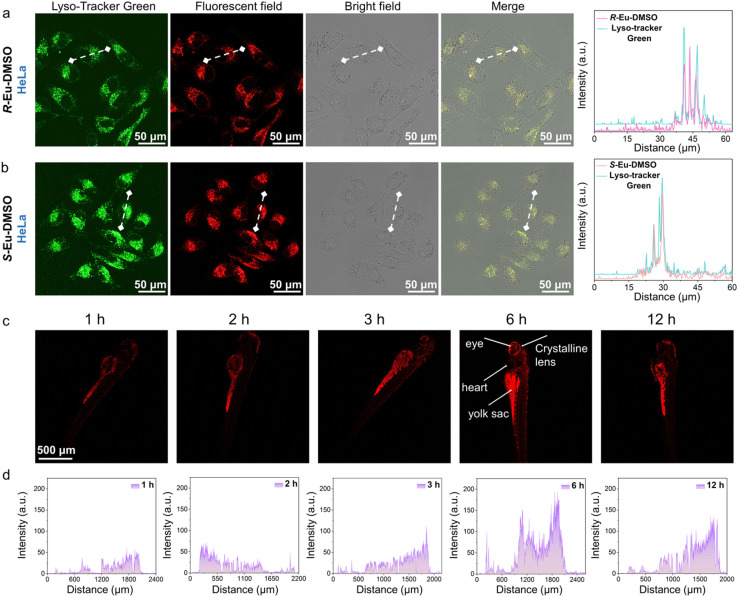
CLSM images of (a) *R*-Eu-DMSO, (b) *S*-Eu-DMSO, and LysoTracker Green (Beyotime Biotechnology) localized on HeLa lysosomes; quantitative analysis of the fluorescence intensity of *R*-Eu-DMSO, *S*-Eu-DMSO, and LysoTracker Green in HeLa cells; (c) CLSM images of *R*-Eu-DMSO co-incubated with zebrafish larvae for 1, 2, 3, 6, and 12 h; (d) quantitative analysis of fluorescence intensity.

Chiral Eu(iii) complexes, as an important class of lanthanide complex emitters, show broad application prospects in the biomedical field due to their unique photophysical properties. However, traditional mononuclear rare earth complexes are susceptible to aggregation-induced quenching of fluorescence due to the easy change in the ligand conformation in solution, and the luminescence of rare earth ions is easily affected by the vibrational quenching effect caused by water or hydroxyl groups. This has, to a certain extent, limited the application of rare earth complex emitters in the field of bioimaging.^61,62^ It is worth noting that rare earth complexes formed by combining AIEgens with rare earth elements have high stability, biocompatibility, and photostability in dilute solutions, which makes up for the shortcomings of traditional mononuclear rare earth complexes in bioimaging applications.^63^ Based on the excellent photophysical properties of *R*/*S*-Eu, their potential for application in bioimaging was further explored. High stability in aqueous solution, low biotoxicity, and highly uniform nanosize are the foundations for their application in cell imaging. Generally, when the absolute value of the zeta potential of particles in a colloid is greater than 20 mV, it indicates that the dispersed system is relatively stable. Therefore, the zeta potentials of *R*/*S*-Eu in a mixed solution (*V*_DMSO_/*V*_water_ = 1/99) were first tested; their absolute values were 39.7 and 26.67 mV, respectively, indicating their high stability in aqueous solution. Furthermore, low cytotoxicity is fundamental to cell imaging. A standardized MTT colorimetric assay was used to assess the cytotoxicity of *R*/*S*-Eu-DMSO against HeLa, MCF-7, MDA-MB-231, HepG2, SK-OV-3, and WI-38 cells. The results showed that after incubation with various concentrations of *R*/*S*-Eu-DMSO solution (0, 6.25, 12.5, 25, 50, and 100 µg mL^−1^), the survival rates of these cells remained above 70%, even with increasing concentrations of the complexes. These indicate that *R*/*S*-Eu-DMSO exhibit negligible cytotoxicity against these cells and can be used for subsequent cell imaging experiments (Fig. 4a, b, S16 and S17). These cells were placed in a 37 °C incubator and allowed to adhere to and grow to a confocal dish density of 90%. *R*/*S*-Eu-DMSO solution was then added to the cells for incubation, and *in vitro* imaging of the cells was performed using two-photon confocal laser scanning microscopy (Fig. 4c). The results demonstrated that *R*/*S*-Eu-DMSO were effectively taken up by the cells and primarily distributed in the cytoplasm of all seven cell types. Quantitative analysis of single-cell fluorescence intensity showed that the fluorescence intensity of *R*-Eu-DMSO in different cells decreased in the order of WI-38 → 4T1 → SK-OV-3 → MDA-MB-231 → HeLa → HepG2 → MCF-7 (Fig. 4d, S18 and S20a–c), and the fluorescence intensity of *S*-Eu-DMSO decreased in the order of 4T1 → SK-OV-3 → HeLa → WI-38 → HepG2 → MCF-7 → MDA-MB-231 (Fig. 4e, S19 and S20d–f), demonstrating that *R*/*S*-Eu-DMSO can effectively label the above seven cell types, and the labeling ability of *R*/*S*-Eu-DMSO on the same cell type is not the same. In addition, we further conducted co-localization imaging experiments between *R*/*S*-Eu-DMSO solutions and a commercial lysosomal probe (Lyso-Tracker Green) (Fig. 5a and b). The experimental results showed that the merged images of *R*/*S*-Eu-DMSO and Lyso-Tracker Green had highly overlapping luminescence signals, demonstrating that both *R*/*S*-Eu-DMSO can specifically label lysosomes.

Zebrafish have become an important vertebrate model for biomedical research due to their high homology with the human genome, transparent early embryos, strong reproductive capacity, and low cost.^64,65^ Based on the excellent photophysical properties and *in vitro* cell imaging performance of *R*-Eu-DMSO, their potential for *in vivo* luminescence imaging in zebrafish was further explored. Specifically, a 50 mg mL^−1^*R*-Eu-DMSO solution was injected into a zebrafish culture medium and incubated for 1, 2, 3, 6, and 12 h. CLSM was then used to evaluate the *in vivo* imaging and distribution of *R*-Eu-DMSO in zebrafish. The experimental results showed that the red luminescence of *R*-Eu-DMSO in zebrafish larvae gradually increased with increasing incubation time. Notably, *R*-Eu-DMSO was primarily concentrated in the liver and yolk sac, while its content was lower in the head and trunk, indicating that *R*-Eu-DMSO can specifically label the yolk sac and liver (Fig. 5c and S21). Furthermore, quantitative analysis of fluorescence intensity was consistent with the experimental results (Fig. 5d). The above results show that lanthanide complexes based on aggregation-induced emission properties show significant advantages in zebrafish imaging, which not only expands the application scope of lanthanide complexes in the field of biological imaging, but also provides new technical means for high-precision biological detection.

## 3 Conclusion

In summary, a pair of dynamic chiral mononuclear Eu(iii) complex isomers (*R*/*S*-Eu) was constructed by improving the conjugation degree of the chiral organic ligand. *R*/*S*-Eu exhibited an aggregation-enhanced antenna effect in aqueous solution *via* a RIR mechanism, enabling high-resolution optical imaging of living cells and zebrafish. Specifically, the dynamic modules within the *R*/*S*-Eu structure in the single-molecule state can rotate freely without obvious emission behavior. After aggregation in glycerol or aqueous solution, the rotation of the molecular rotors in the *R*/*S*-Eu structure is restricted by hydrogen bonds and steric hindrance and cannot rotate freely, resulting in bright red emission. DFT calculations show that the HOMO energy level of ligand L2 closely matches the LUMO energy level of *R*-Eu, ensuring a highly efficient antenna effect. The chiral isomers *R*/*S*-Eu exhibit mirror-symmetrical CPL emission peaks at 542, 573, and 581 nm, respectively, attributable to the characteristic fingerprint emission of Eu(iii) ions. Both *R*-Eu-DMSO and *S*-Eu-DMSO demonstrated high-resolution optical imaging of various living cell types, and co-localization experiments demonstrated their specific localization to lysosomal organelles. When co-incubated with zebrafish, *R*-Eu-DMSO was primarily distributed in the yolk sac and liver, exhibiting bright red luminescence. This work not only provides new insights into the construction of multifunctional chiral lanthanide complexes but also effectively expands the optical imaging applications of chiral lanthanide complexes in aqueous systems.

## Author contributions

Z.-H. Zhu and H.-L. Wang conceived the study. R.-Y. Li and M.-J. Tang carried out detailed studies. R.-Y. Li, M.-J. Tang, H.-L. Wang, Z.-H. Zhu, Y.-L. Li, F. Yang, and H.-H. Zou analyzed the problem and designed the method. R.-Y. Li, M.-J. Tang, H.-L. Wang, Z.-H. Zhu, Y.-L. Li, and F. Yang co-analyzed the results. R.-Y. Li wrote the manuscript and Z.-H. Zhu and H.-L. Wang made modifications. Z.-H. Zhu and H.-L. Wang provided strategic guidance. All authors contributed to useful discussions.

## Conflicts of interest

There are no conflicts to declare.

## Supplementary Material

SC-OLF-D5SC09594H-s001

SC-OLF-D5SC09594H-s002

## Data Availability

The data that support the findings of this study are available from the corresponding author upon reasonable request. CCDC 2479516 and 2495269 contain the supplementary crystallographic data for this paper.^66*a*,*b*^ Supplementary information (SI): experimental section, and additional tables and figures. See DOI: https://doi.org/10.1039/d5sc09594h.
