# Supplementary material for: Improving the conjugation of organic ligands enhances the antenna effect and promotes the luminescence and optical imaging of chiral mononuclear Eu(iii) complexes
Source: Chem Sci. 2025 Dec 26;17(8):4157–67. doi: 10.1039/d5sc09594h (PMC12776613; doi:10.1039/d5sc09594h)
Supplement: SC-017-D5SC09594H-s001 [file SC-017-D5SC09594H-s001.pdf]

Supplementary Information (SI) for Chemical Science.
This journal is © The Royal Society of Chemistry 2025

Supporting Information

Improving the conjugation of organic ligands enhances the antenna effect and promotes the luminescence and optical imaging of chiral mononuclear Eu(III) complexes

Ru-Yan Li,[a,+] Meng-Juan Tang,[b,+] Yun-Lan Li,[b] Fan Yang,[b] Hua-Hong Zou,[b] Hai-Ling Wang,[a,] and Zhong-Hong Zhu,[a,]**

[a]School of Chemistry and Chemical Engineering, Guangxi Key Laboratory of Electrochemical Energy Materials, Guangxi University, Nanning, 530004, P. R. China

[b]Key Laboratory for Chemistry and Molecular Engineering of Medicinal Resources (Ministry of Education of China), Guangxi Key Laboratory of Chemistry and Molecular Engineering of Medicinal Resources, School of Chemistry and Pharmaceutical Sciences, Guangxi Normal University, Guilin 541004, P. R. China

**E-mail (Corresponding author): whling@gxu.edu.cn (H.-L. Wang), zzhong@gxu.edu.cn (Z.-H. Zhu).*

Table of Contents:

Supporting Tables	
**Table S1**	Crystallographic data of ***R/S*-Eu**.
**Table S2**	Statistical report on this work and the g_{lum} values of lanthanide complexes.
**Table S3**	Selected bond lengths (Å) and angles (°) of ***R*-Eu**.
**Table S4**	Selected bond lengths (Å) and angles (°) of ***S*-Eu**.
**Table S5**	*SHAPE* analysis of the Eu(III) ion in ***R*-Eu**.
**Table S6**	*SHAPE* analysis of the Eu(III) ion in ***S*-Eu**.
**Supporting Figures**	
**Figure S1**	(a) Stacking structure formed by four different hydrogen bonds between independent ***R*-Eu** units; (b) Stacking structure formed by four different hydrogen bonds between independent ***S*-Eu** units; (c and d) Coordination structure of the metal-centered Eu(III) ion in ***R*/*S*-Eu**.
**Figure S2**	EDS Mapping images of ***S*-Eu**.
**Figure S3**	Powder X-ray diffraction pattern (PXRD) of ***R/S*-Eu**.
**Figure S4**	FT-IR absorption spectra of ***R*/*S*-Eu** and ***R*/*S*-L2**.
**Figure S5**	Thermogravimetric curves (TG) of ***R/S*-Eu**.
**Figure S6**	(a and b) Solid-state luminescence spectra of ***R*/*S*-Eu**; (c and d) Luminescence spectra of ***R*/*S*-Eu** dispersed in DMSO.
**Figure S7**	Decay curves of ***R/S*-Eu**.
**Figure S8**	Quantum yields of ***R/S*-Eu-DMSO**.
**Figure S9**	The excitation-dependent three-dimensional emission spectra of ***R*/*S*-Eu-DMSO** (a and b) in DMSO solution, respectively; CPL spectra (c), DC spectra (c), and g_{lum} value (d) of ***R*/*S*-Eu** dispersed in $CHCl_3$ solution, respectively.
**Figure S10**	(a) Solid-state UV-Vis absorption spectra of complexes ***R/S*-Eu**; UV-vis absorption spectra of complexes ***R*-Eu** (b) and ***S*-Eu** (c) in mixed solutions of Gly/DMSO with different f_w contents.
**Figure S11**	(a) Emission spectra of ***R/S*-Eu** in different Gly/MeOH mixed solutions under 395 nm excitation; (c) Emission spectra of ***R/S*-Eu** in different Gly/DMF mixed solutions under 395 nm excitation; (b and d) Luminescence intensity of ***R/S*-Eu** at 617 nm as a function of f_w.
**Figure S12**	HRESI-MS spectrum of ***R*-Eu-DMSO** in positive ion mode (MeOH was used as the mobile phase), and comparison of experimental (black) and simulated (red) values of the main molecular ion peaks.

**Figure S13**	HRESI-MS spectrum of ***S*-Eu-DMSO** in positive ion mode (MeOH was used as the mobile phase), and comparison of experimental (black) and simulated (red) values of the main molecular ion peaks.
**Figure S14**	HRESI-MS spectrum of ***R*-Eu-DMSO** (MeCN was used as the mobile phase) in positive ion mode and comparison of experimental (black) and simulated (red) values of the main molecular ion peaks.
**Figure S15**	Comparison of experimental (black) and simulated (red) values of the main molecular ion peaks of the HRESI-MS spectrum of ***S*-Eu-DMSO** in positive ion mode (MeCN was used as the mobile phase).
**Figure S16**	Growth inhibition rate of HeLa (a), MDA-MB-231 (b), HepG2 (c), and SK-OV-3 (d) cells by different concentrations of ***R*-Eu-DMSO-DMSO**.
**Figure S17**	Growth inhibition rate of WI-38 (a) cells by different concentrations of ***R*-Eu-DMSO**; Growth inhibition rate of MDA-MB-231 (b), HeLa (c), and HepG2 (d) cells by different concentrations of ***S*-Eu-DMSO**.
**Figure S18**	CLSM images of ***R*-Eu-DMSO** after co-incubation with HeLa (a), HepG2 (b), SK-OV-3 (c), and 4T1 (d).
**Figure S19**	CLSM images of ***S*-Eu-DMSO** after co-incubation with HeLa (a), MDA-MB-231 (b), SK-OV-3 (c), and 4T1 (d).
**Figure S20**	CLSM images and quantitative analysis of fluorescence intensity of ***R*-Eu-DMSO** after co-incubation with MCF-7 (a), MDA-MB-231 (b), and WI-38 (c); CLSM images and quantitative analysis of fluorescence intensity of ***S*-Eu-DMSO** after co-incubation with HepG2 (d), MCF-7 (e), and WI-38 (f).
**Figure S21**	CLSM images of zebrafish co-incubated with ***R*-Eu-DMSO** for 1, 2, 3, 6, and 12 h, respectively.

Experimental Section

Materials and Measurements

All reagents were obtained from commercial sources and used without further purification. Transmission electron microscopy (TEM) images and energy dispersive spectroscopy (EDS) elemental maps were recorded with a ThermoFisher Scientific Talos F200S transmission electron microscope. Powder X-ray diffraction (PXRD) spectra were measured at 298 K (Mo-Kα) using a Bruker SMART CCD diffractometer. The infrared absorption spectra were recorded using a PE Spectrum FT-IR spectrometer (400-4000 cm^{-1}) with KBr containing approximately 0.5%. Thermogravimetric analysis (TGA) was performed using a NETZSCH TG 209 F3 in flowing nitrogen at a heating rate of 5 °C/min. UV-vis absorption spectra were recorded using a Shimadzu UV-2600 spectrophotometer. The circular dichroism (CD) spectra were measured at room temperature using a JASCO J-1500 polarization spectrometer. The absorbance of 3-(4,5-dimethylthiazol-2-yl)-2,5-diphenyltetrazolium bromide (MTT) was measured using the Cytation 5 Multifunctional Detection System. Cell imaging, zebrafish imaging, and colocalization were recorded using a TCS SPB DIVE two-photon laser confocal microscope.

Single-crystal X-ray crystallography

Diffraction data for the complex was collected on a Bruker SMART CCD diffractometer (Cu-Kα radiation and λ = 1.54 Å) in Φ and ω scan modes. The structures were solved by direct methods, followed by difference Fourier syntheses, and then refined by full-matrix least-squares techniques on F^2 using *SHELXL*. All other non-hydrogen atoms were refined with anisotropic thermal parameters. Hydrogen atoms were placed at calculated positions and isotopically refined using a riding model. Table S1 summarizes X-ray crystallographic data and refinement details for the complexes. The CCDC reference numbers for the ***R/S*-Eu** crystal structures are 2479516 and 2495269, respectively.

Synthesis of *R*-Eu

(1*R*,2*R*)-1,2-diphenylethylenediamine (0.25 mmol, 0.053 g) and 2-pyridinecarboxaldehyde (0.5 mmol, 48 μL) were dissolved in 10 mL of CH_3OH and stirred for 1 h. $Eu(NO_3)_3 \cdot 6H_2O$ (0.25 mmol, 0.1115 g) was added, followed by 10 mL of CH_3OH. The mixture was evaporated at room temperature to

afford colorless blocky crystals. Infrared spectral data (IR, KBr, cm^{-1}): 3462 (s), 1595 (s), 1386 (s), 1120 (w), 767 (m), 700 (m), 617 (m).

Synthesis of *S*-Eu

The synthesis method was similar to that for ***R*-Eu** by using (1*S*,2*S*)-1,2-diphenylethylenediamine instead of (1*R*,2*R*)-1,2-diphenylethylenediamine. Infrared spectral data (IR, KBr, cm^{-1}): 3465 (s), 1586 (s), 1383 (s), 1121 (w), 766 (m), 699 (m), 615 (m).

MTT assay of *R/S*-Eu-DMSO

The cytotoxicity of ***R/S*-Eu-DMSO** against HeLa, MCF-7, WI-38, HepG2, MDA-MB-231, and SK-OV-3 cells was investigated using the MTT assay. Cells were grown adherently in a 37 °C, 5% CO_2 incubator. Cells were incubated with various concentrations of ***R/S*-Eu-DMSO**. Cell viability was calculated based on MTT absorbance.

Cell imaging of *R/S*-Eu-DMSO

HeLa, MCF-7, WI-38, HepG2, MDA-MB-231, 4T1, and SK-OV-3 cells were cultured in 20 mm glass-bottomed culture dishes at 37 °C in a humidified atmosphere with 5% CO_2. After cell growth reached 90% confluence, the cells were washed three times with PBS. A 20 mg/mL solution of ***R/S*-Eu-DMSO** was then added to the culture dishes (final concentration: 25 μg/mL). After 16 h of incubation, the dishes were removed, washed three times with PBS, and CLSM images were acquired.

Zebrafish imaging of *R*-Eu-DMSO

Zebrafish were cultured in a petri dish at room temperature. ***R*-Eu-DMSO** solution was then added to the dish to a final concentration of 25 μg/mL. After incubation for 1, 2, 3, 6, and 12 hours, the zebrafish were washed three times, and CLSM images were acquired.

Table S1. Crystallographic data of ***R/S*-Eu**.

Complexes	***R*-Eu**	***S*-Eu**
Formula	$2(C_{26}H_{22}Eu_1N_7O_9)$	$2(C_{26}H_{22}Eu_1N_7O_9)$
Formula weight	1456.94	1456.94
T (K)	100	100
Crystal system	triclinic	triclinic
Space group	$P1$	$P1$
a (Å)	9.5245(2)	9.5275(2)
b (Å)	12.6520(3)	12.6485(3)
c (Å)	12.7210(3)	12.7241(4)
α (°)	75.746(2)	75.718(3)
β (°)	68.755(2)	68.740(3)
γ (°)	77.174(2)	77.160(2)
V (Å^3)	1369.73(6)	1369.80(7)
Z	1	1
Reflns coll.	11339	7089
Unique reflns	20609	17689
R_{int}	0.043	0.041
$^aR_1[I \geq 2\sigma(I)]$	0.032	0.071
$^bwR_2$(all data)	0.100	0.179
GOF	0.753	1.039

$^aR_1=\Sigma||F_o|-|F_c||/\Sigma|F_o|$, $^bwR_2 = \Sigma[w(F_o^2-F_c^2)^2]/\Sigma[w(F_o^2)^2]^{1/2}$

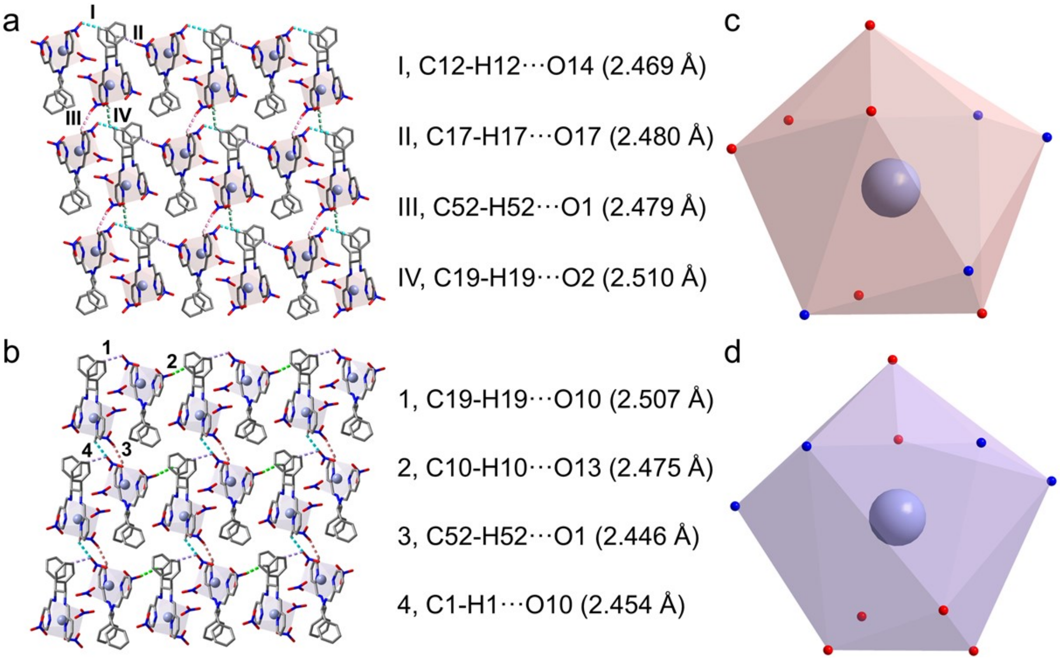

Figure S1. (a) Stacking structure formed by four different hydrogen bonds between independent ***R*-Eu** units; (b) Stacking structure formed by four different hydrogen bonds between independent ***S*-Eu** units; (c and d) Coordination structure of the metal-centered Eu(III) ion in ***R*/*S*-Eu**.

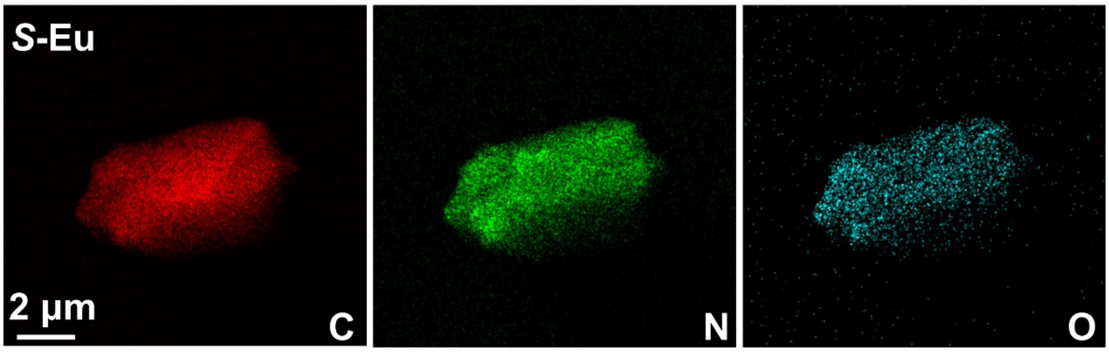

Figure S2. EDS Mapping images of ***S*-Eu**.

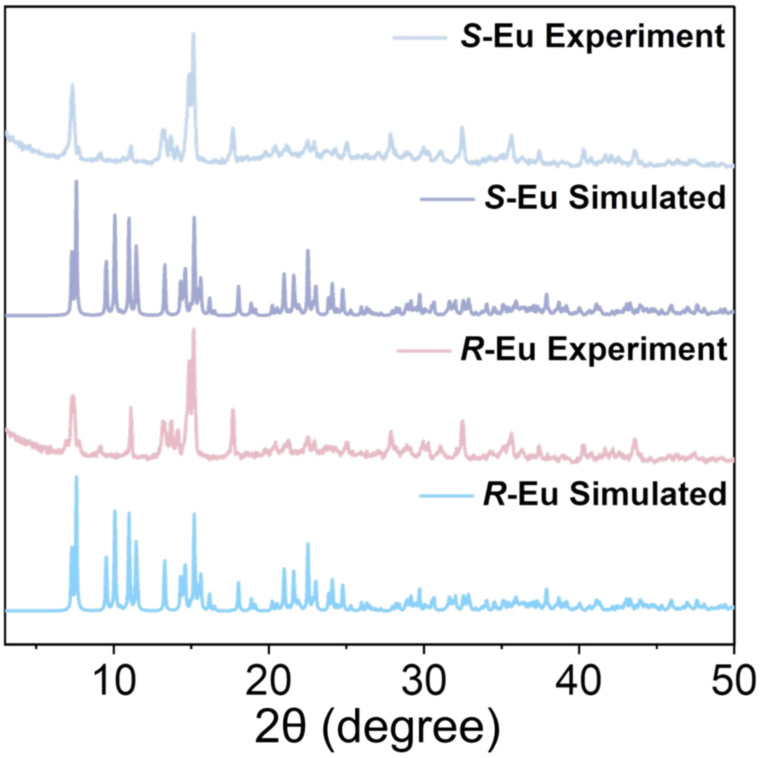

Figure S3. Powder X-ray diffraction pattern (PXRD) of ***R/S*-Eu**.

Fourier transform infrared (FT-IR) analysis

The FT-IR absorption peak in the range of 3200~3600 cm^{-1} can be attributed to the stretching vibration of the O-H bond. Compared with the ligand, the above absorption peak of the complex is wider and the overall "bulge" is more obvious. This is because the presence of hydrogen bonding and intermolecular forces in the complex causes the peak shape of the vibration absorption to change. The absorption peak in the range of 1580~1650 cm^{-1} belongs to the absorption peak of C=N stretching vibration. The absorption peak undergoes displacement and deformation in the complex, and the peak intensity also decreases, which indicates that the nitrogen atoms in the ligand coordinate with the Eu(III) ions, changing the electron cloud density and force constant of the C=N chemical bond. The absorption peak in the range of 690-710 cm^{-1} can be attributed to the vibration of the pyridine ring. After the ligand coordinates with the Eu(III) ions, the chemical environment of the pyridine ring is changed, and the obvious absorption peak at 699 cm^{-1} disappears.

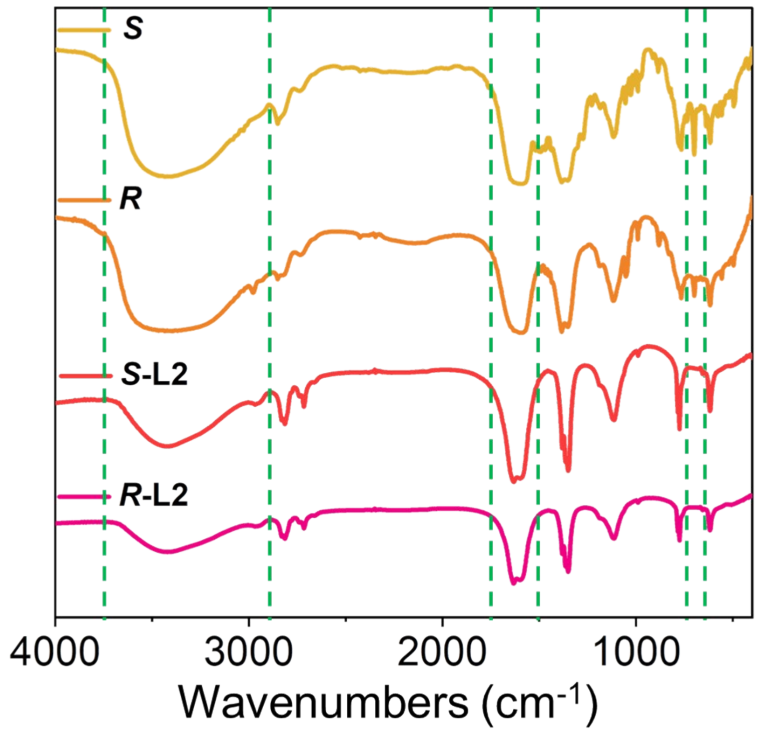

Figure S4. FT-IR absorption spectra of ***R*/*S*-Eu** and ***R*/*S*-L2**.

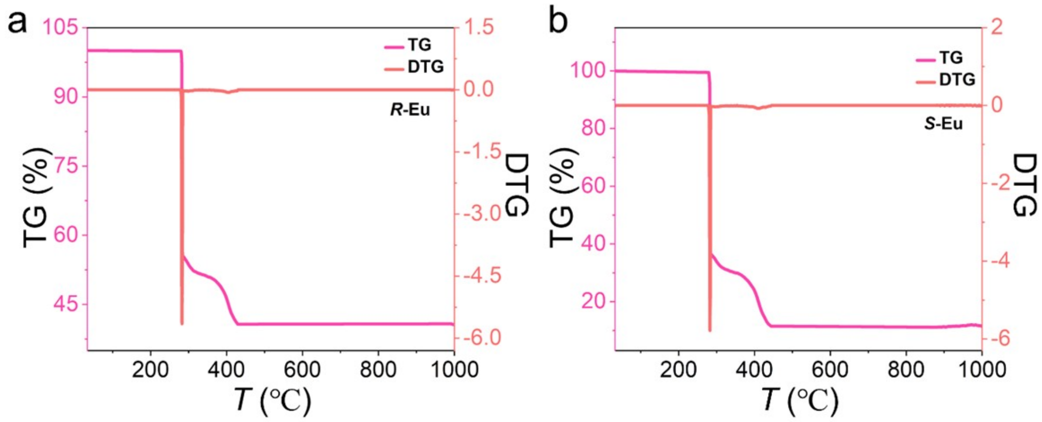

Figure S5. Thermogravimetric curves (TG) of ***R/S*-Eu**.

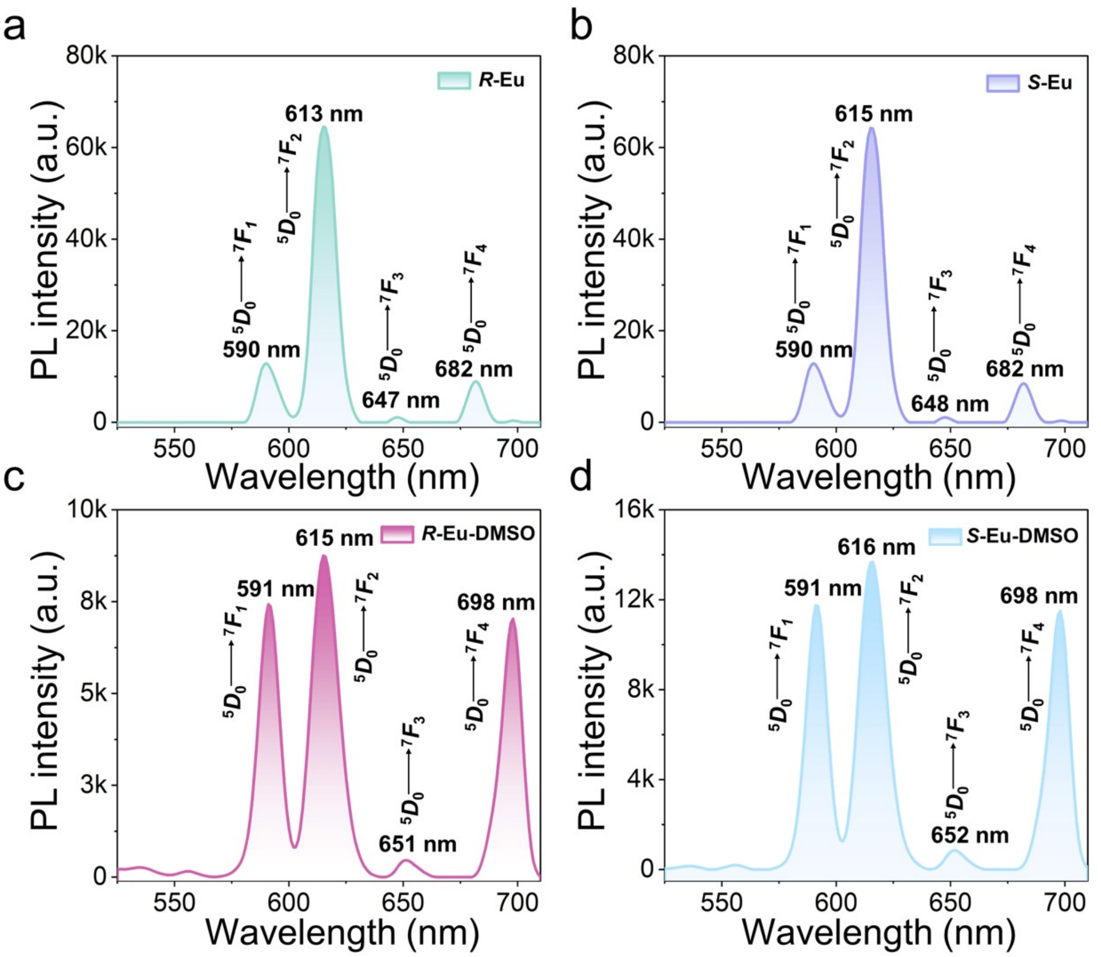

Figure S6. (a and b) Solid-state luminescence spectra of ***R*/*S*-Eu**; (c and d) Luminescence spectra of ***R*/*S*-Eu** dispersed in DMSO.

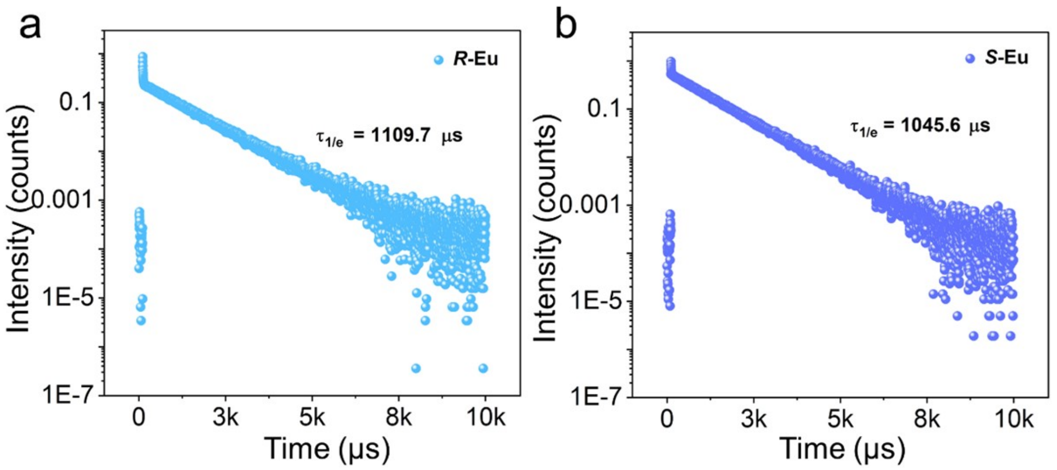

Figure S7. Decay curves of ***R/S*-Eu**.

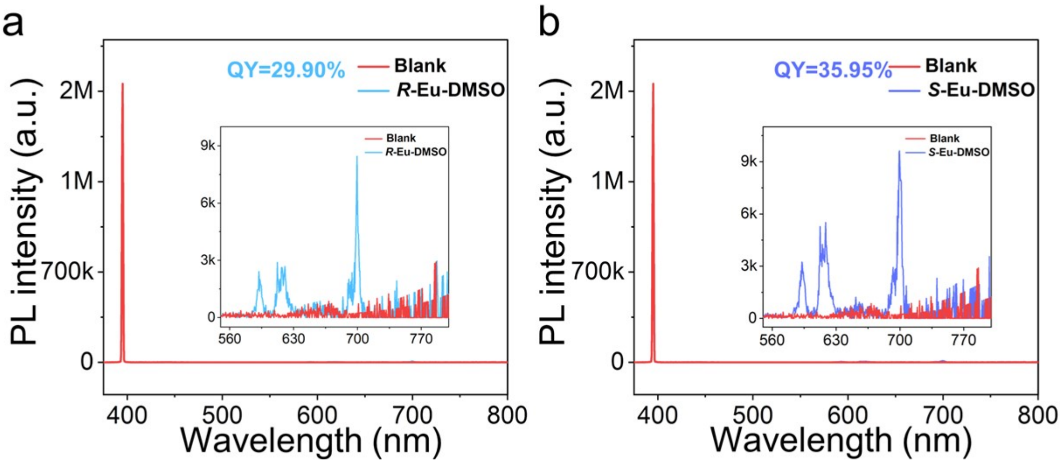

Figure S8. Quantum yields of ***R/S*-Eu-DMSO**.

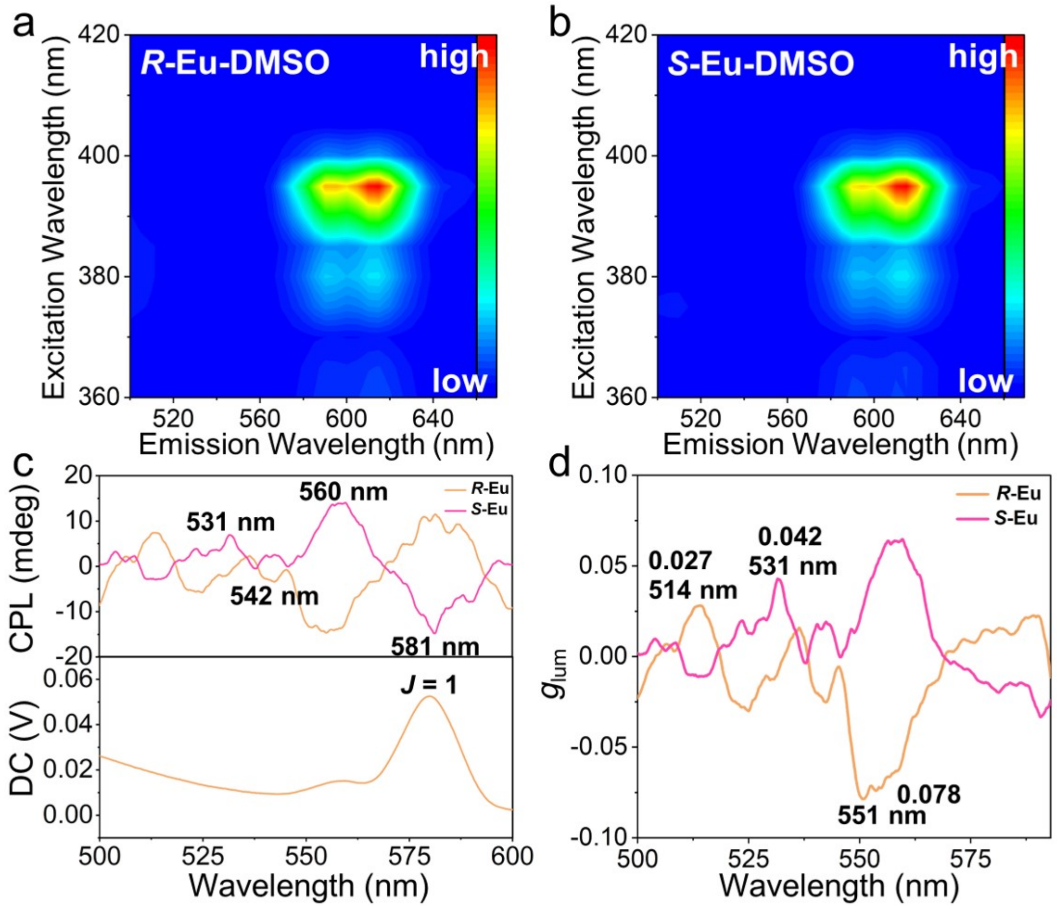

Figure S9. The excitation-dependent three-dimensional emission spectra of ***R*/*S*-Eu-DMSO** (a and b) in DMSO solution, respectively; CPL spectra (c), DC spectra (c), and g_{lum} value (d) of ***R*/*S*-Eu** dispersed in $CHCl_3$ solution, respectively.

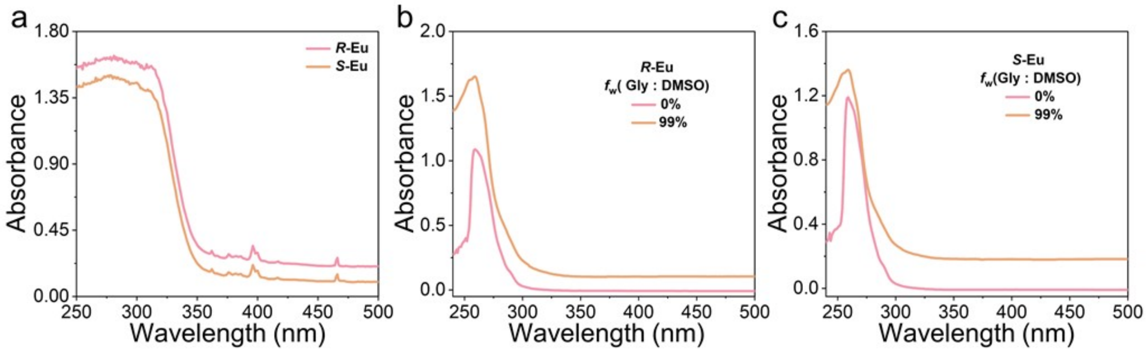

Figure S10. (a) Solid-state UV-Vis absorption spectra of complexes ***R/S*-Eu**; UV-Vis absorption spectra of complexes ***R*-Eu** (b) and ***S*-Eu** (c) in mixed solutions of Gly/DMSO with different f_{w} contents.

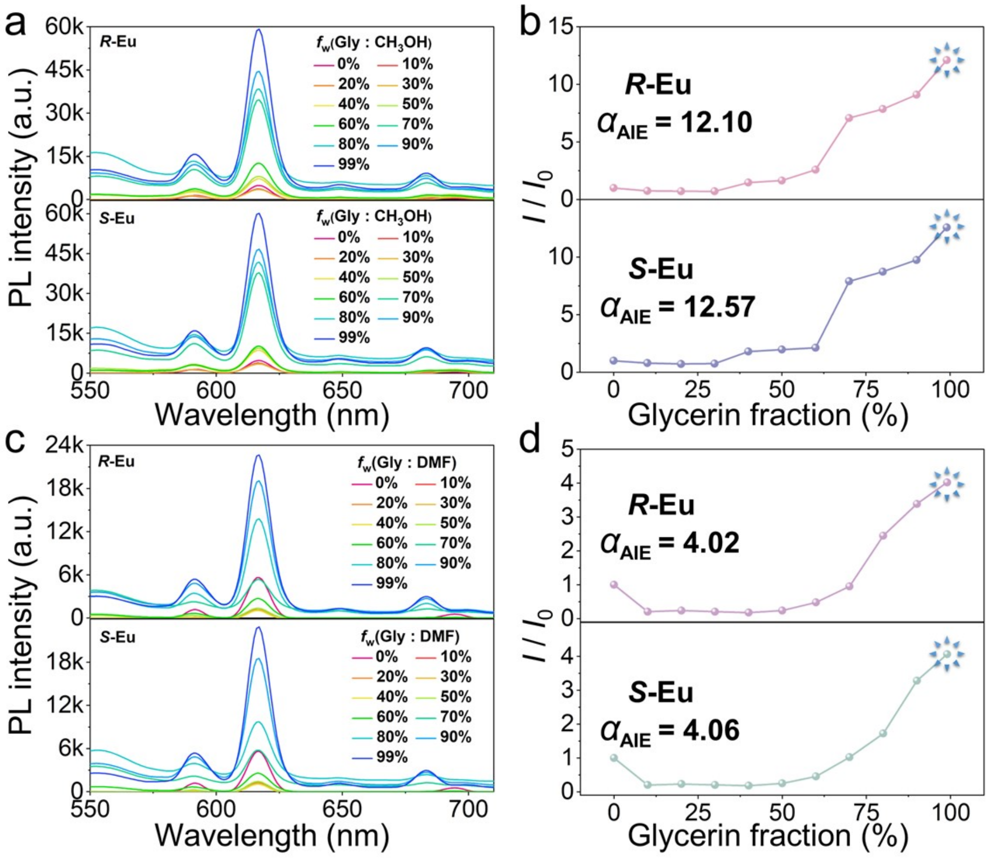

Figure S11. (a) Emission spectra of ***R/S*-Eu** in different Gly/MeOH mixed solutions under 395 nm excitation; (c) Emission spectra of ***R/S*-Eu** in different Gly/DMF mixed solutions under 395 nm excitation; (b and d) Luminescence intensity of ***R/S*-Eu** at 617 nm as a function of f_{w}.

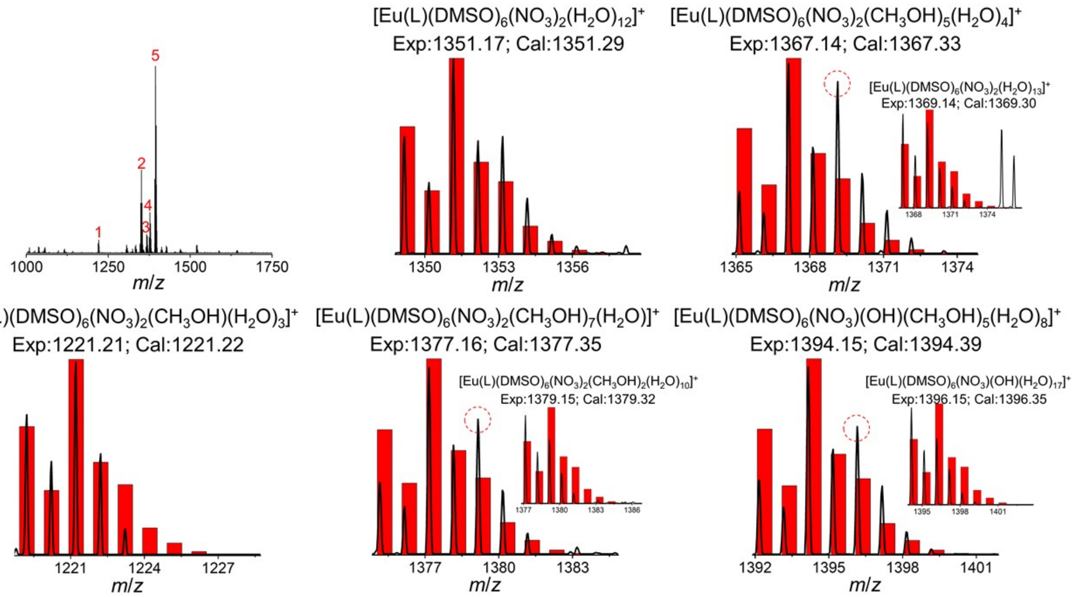

Figure S12. HRESI-MS spectrum of ***R*-Eu-DMSO** in positive ion mode (MeOH was used as the mobile phase), and comparison of experimental (black) and simulated (red) values of the main molecular ion peaks.

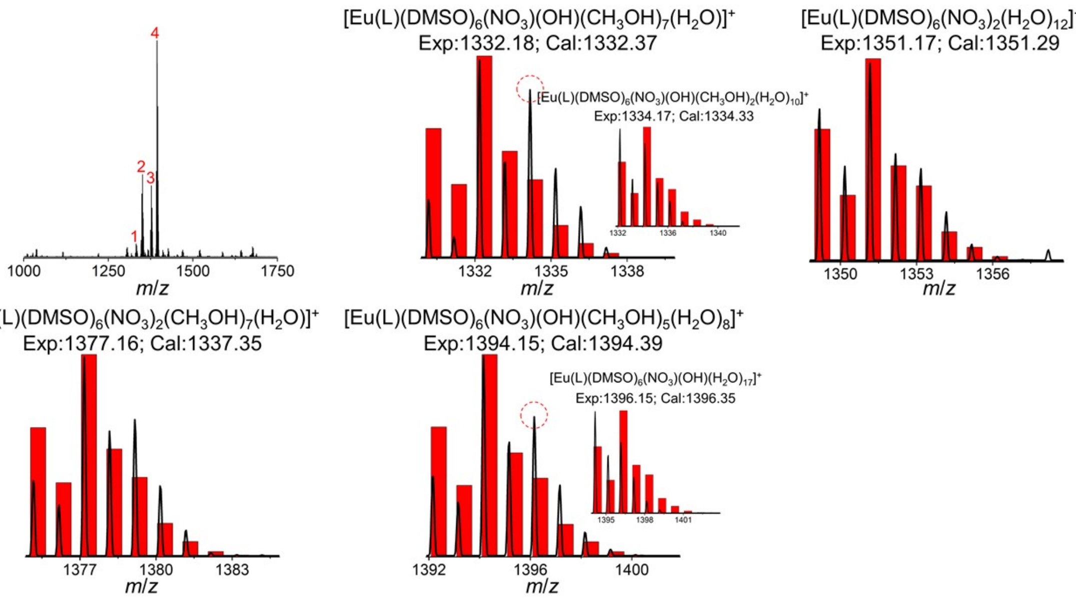

Figure S13. HRESI-MS spectrum of ***S*-Eu-DMSO** in positive ion mode (MeOH was used as the mobile phase), and comparison of experimental (black) and simulated (red) values of the main molecular ion peaks.

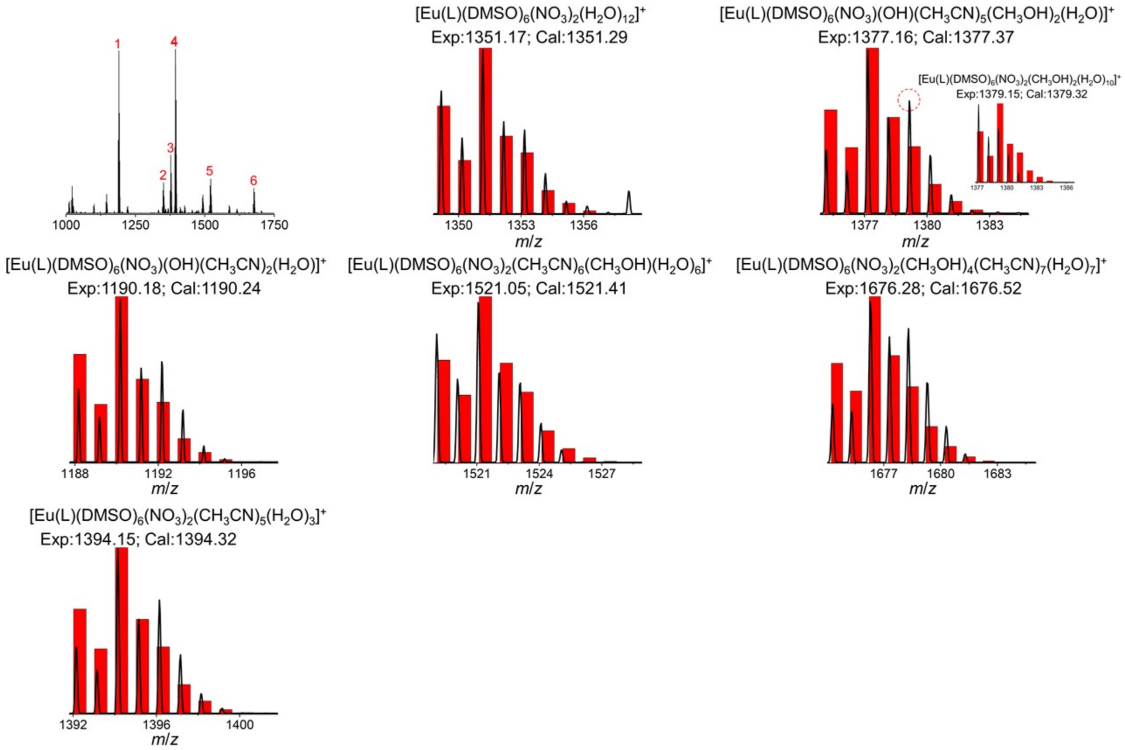

Figure S14. HRESI-MS spectrum of ***R*-Eu-DMSO** (MeCN was used as the mobile phase) in positive ion mode and comparison of experimental (black) and simulated (red) values of the main molecular ion peaks.

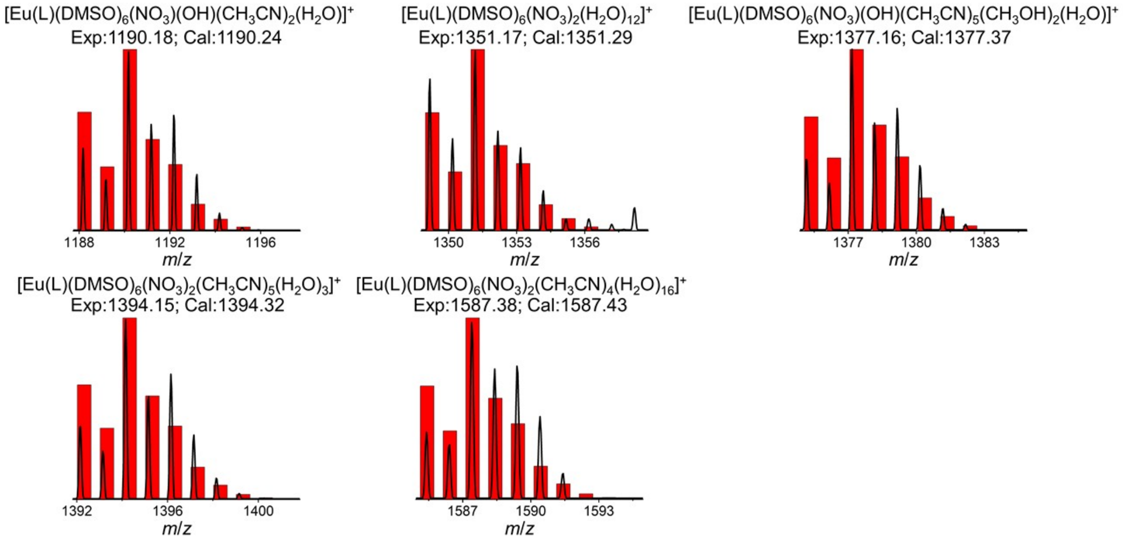

Figure S15. Comparison of experimental (black) and simulated (red) values of the main molecular ion peaks of the HRESI-MS spectrum of ***S*-Eu-DMSO** in positive ion mode (MeCN was used as the mobile phase).

Table S2. Statistical report on this work and the g_{lum} values of lanthanide complexes.

Numbers	**Cluster core structure**	**Molecular formula**	$\|g_{lum}\|$	**Journals published**
1	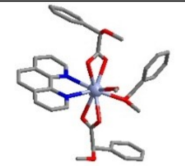	$EuC_{39}N_2O_8$	0.013	*Dalt. Trans.*, 2023, **52**, 1122-1132.
2	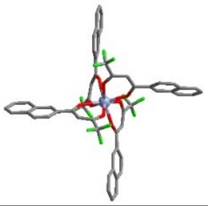	$EuC_{56}O_8F_{12}$	0.02	*Inorg. Chem. Front.*,2025.
3	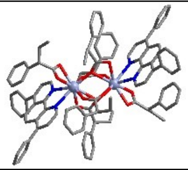	$Eu_2C_{108}N_4O_{12}$	0.02	*Dalt. Trans.*, 2024, **53**, 13566-13582.
4	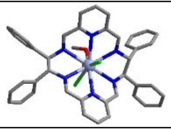	$EuC_{43}N_6OCl_2$	0.084	*Rare Met.*, 2025.
5	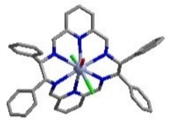	$EuC_{42}N_6OCl_2$	0.098	*Inorg. Chem. Front.*, 2024, **11**, 2039-2048.
6	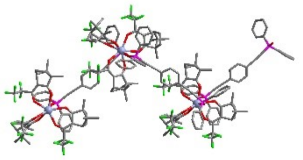	$Eu_3C_{219}O_{24}F_{45}P_6$	0.10	*Dalt. Trans.*, 2022, **52**, 796-805.
7	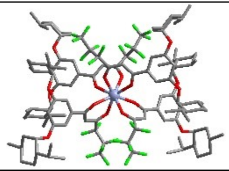	$EuC_{128}O_{16}F_{28}$	0.15	*Inorg. Chem. Front.*, 2025, **12**, 1176-1186.
8	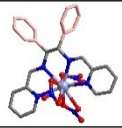	$C_{26}H_{22}Eu_1N_7O_9$	0.159	This work
9	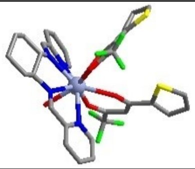	$EuC_{34}N_4O_5F_6S_2$	0.2	*Inorg. Chem.*, 2018, **57**, 10257-10264.
10	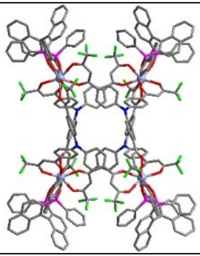	$Eu_4C_{296}P_8N_4O_{32}F_{36}$	0.2	*J. Am. Chem. Soc.*, 2019, **141**, 19634-19643.

11	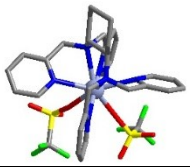	$TbC_{32}N_6O_6F_6S_2$	0.22	*Inorg. Chem.*, 2020, **59**, 7657-7665.
12	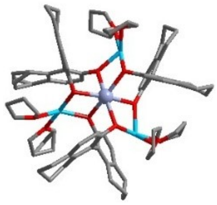	$TbNa_3C_{87}O_{12}$	0.32	*Chem. Commun.*, 2020, **56**, 14813-14816.
13	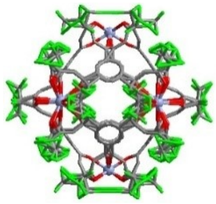	$Tb_6C_{336}O_{96}F_{240}$	0.53	*Angew. Chem. Int. Ed.*, 2025, **64**, e202421426.
14	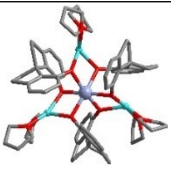	$TbNa_3C_{75}O_{12}$	0.53	*J. Am. Chem. Soc.*, 2022, **144**, 22421-22425.
15	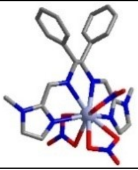	$EuC_{24}N_9O_9$	0.64	*Nat. Commun.*, 2024, **15**, 2896.
16	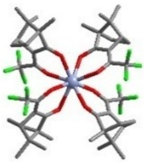	$EuC_{48}O_8F_{12}$	1.54	*Angew. Chem. Int. Ed.*, 2024, **63**, e202405584

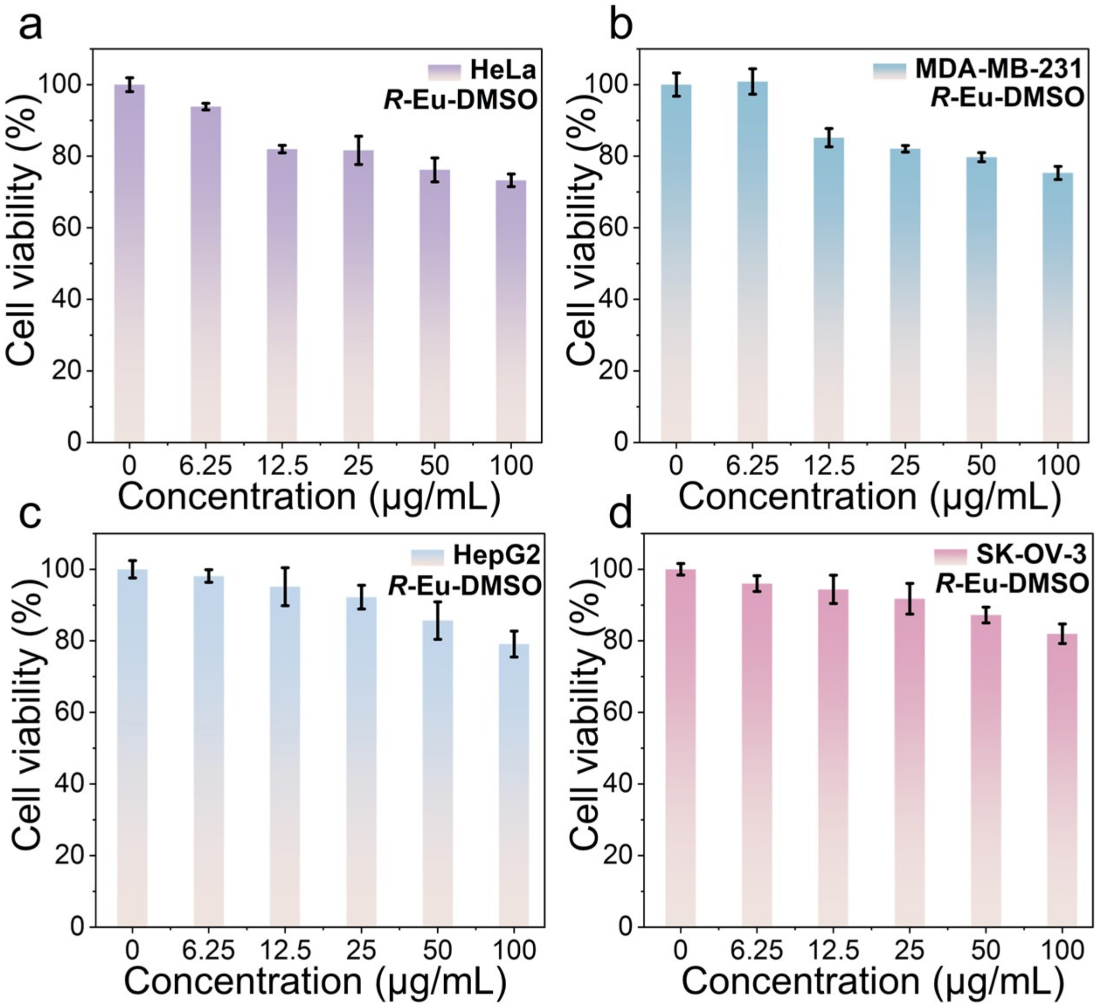

Figure S16. Growth inhibition rate of HeLa (a), MDA-MB-231 (b), HepG2 (c), and SK-OV-3 (d) cells by different concentrations of ***R*-Eu-DMSO**.

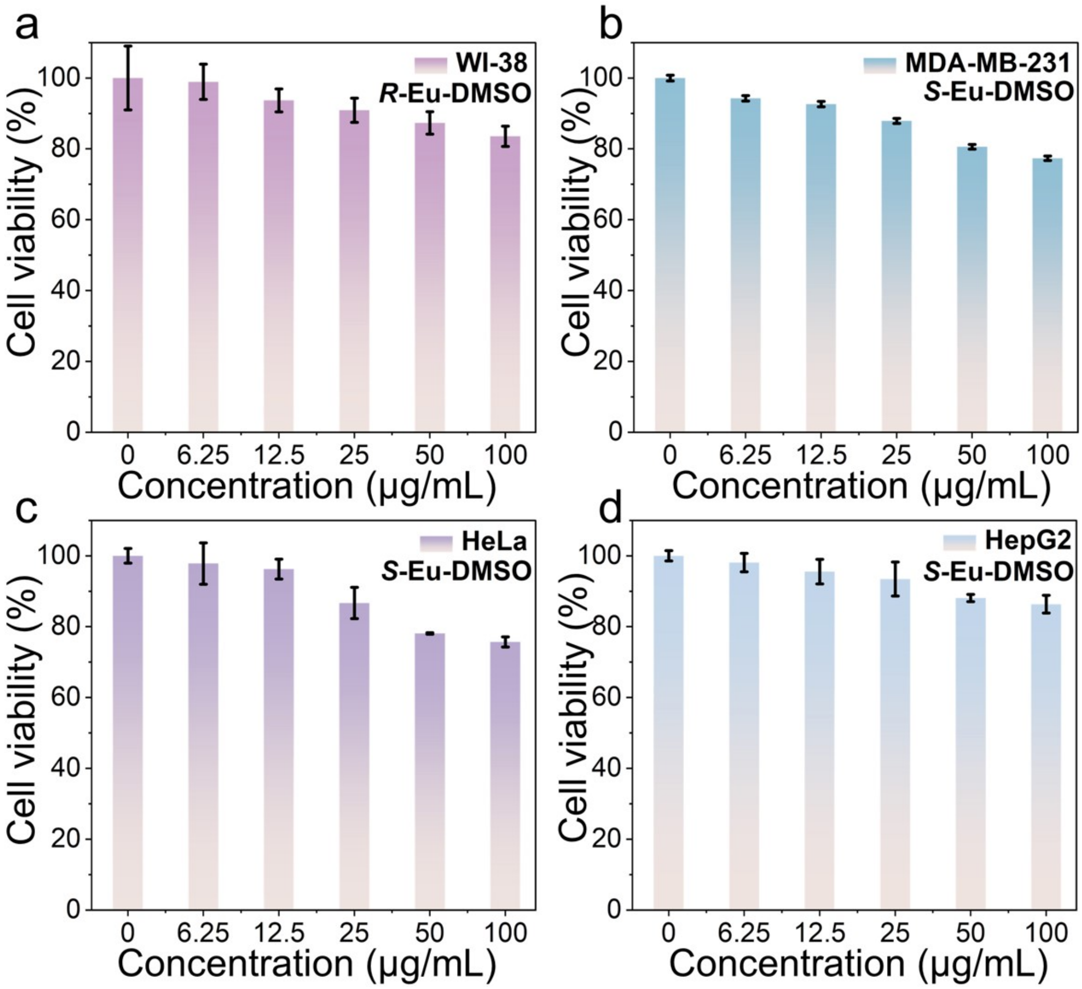

Figure S17. Growth inhibition rate of WI-38 (a) cells by different concentrations of ***R*-Eu-DMSO**; Growth inhibition rate of MDA-MB-231 (b), HeLa (c), and HepG2 (d) cells by different concentrations of ***S*-Eu-DMSO**.

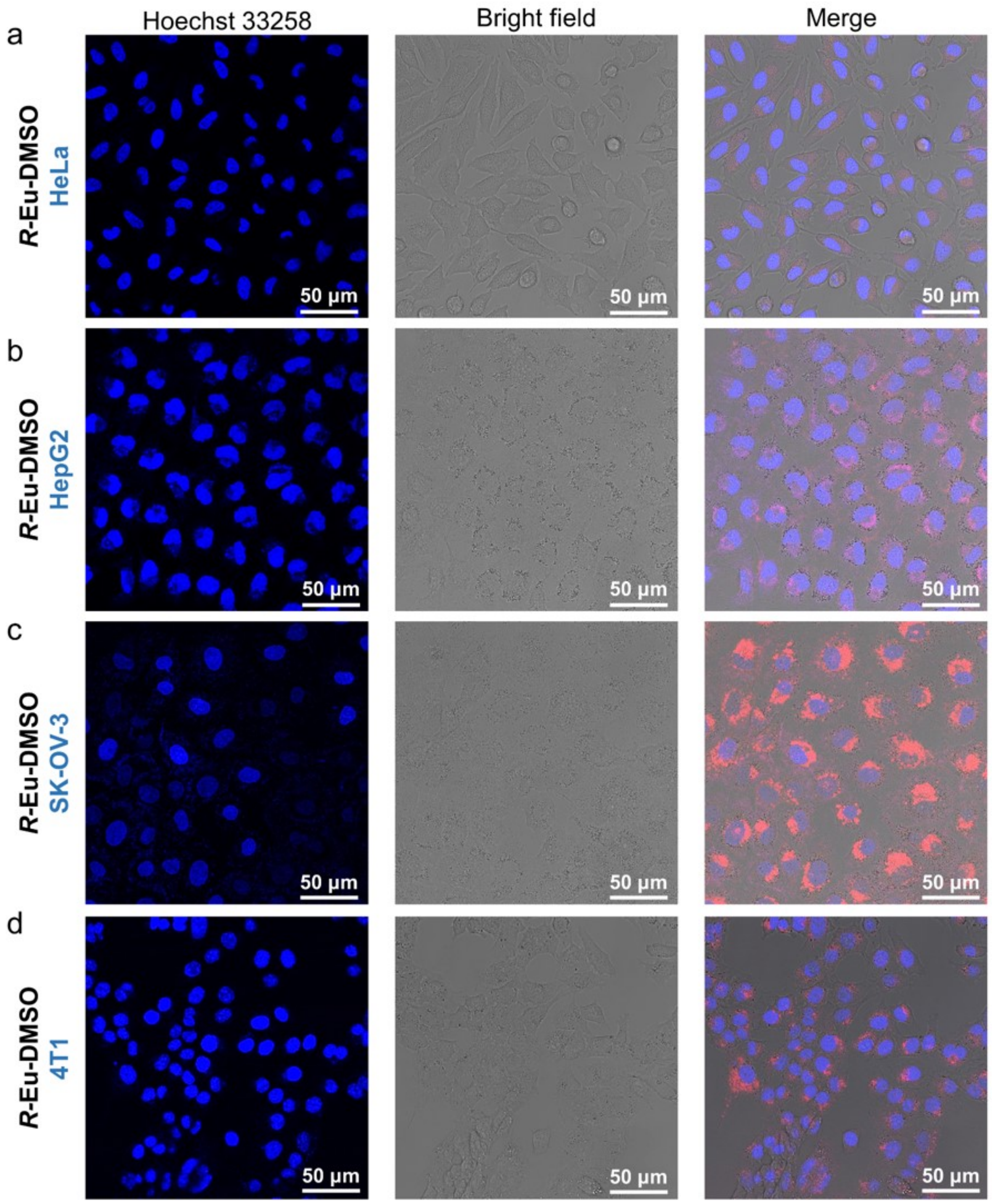

Figure S18. CLSM images of ***R*-Eu-DMSO** after co-incubation with HeLa (a), HepG2 (b), SK-OV-3 (c), and 4T1 (d).

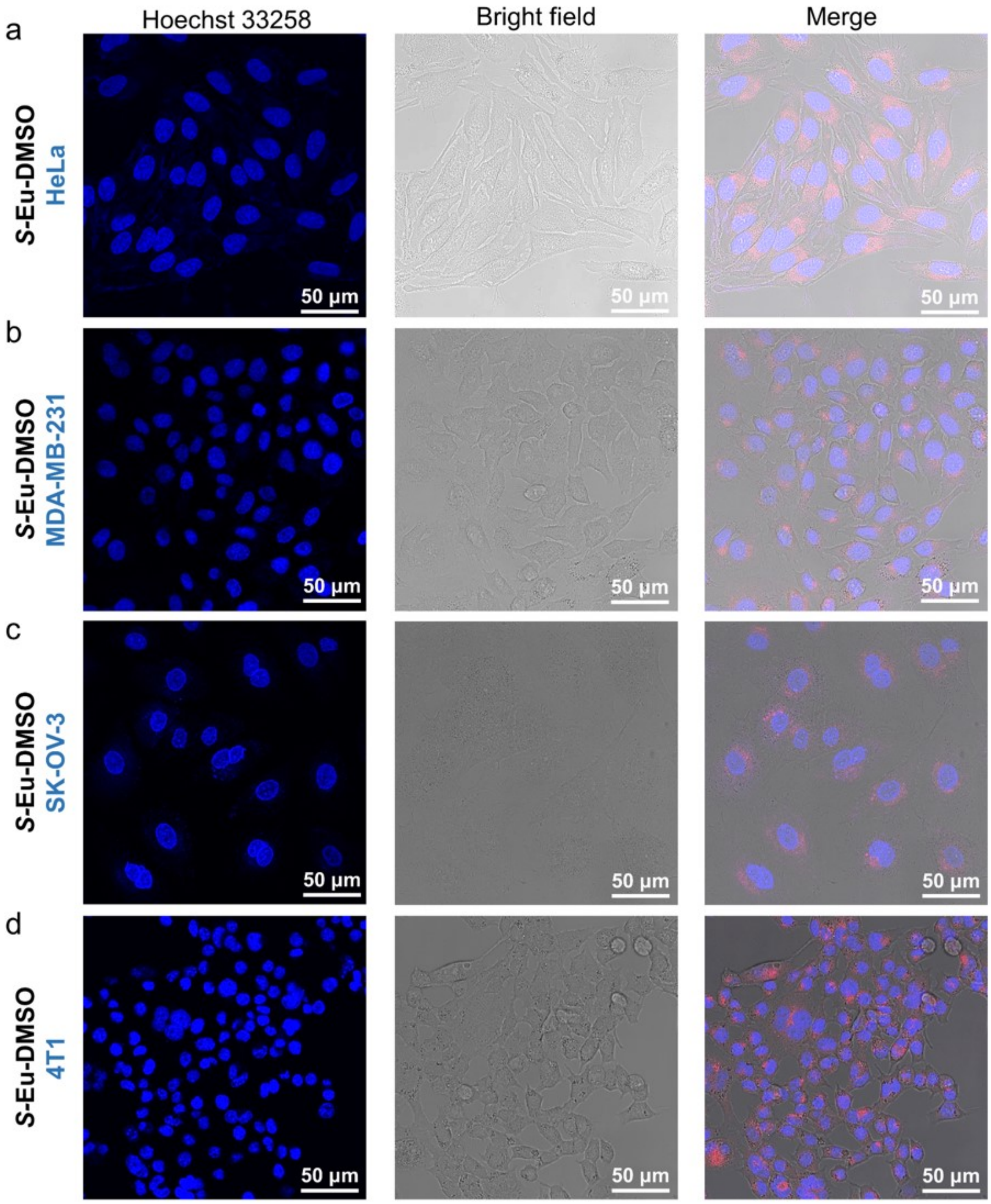

Figure S19. CLSM images of ***S*-Eu-DMSO** after co-incubation with HeLa (a), MDA-MB-231 (b), SK-OV-3 (c), and 4T1 (d).

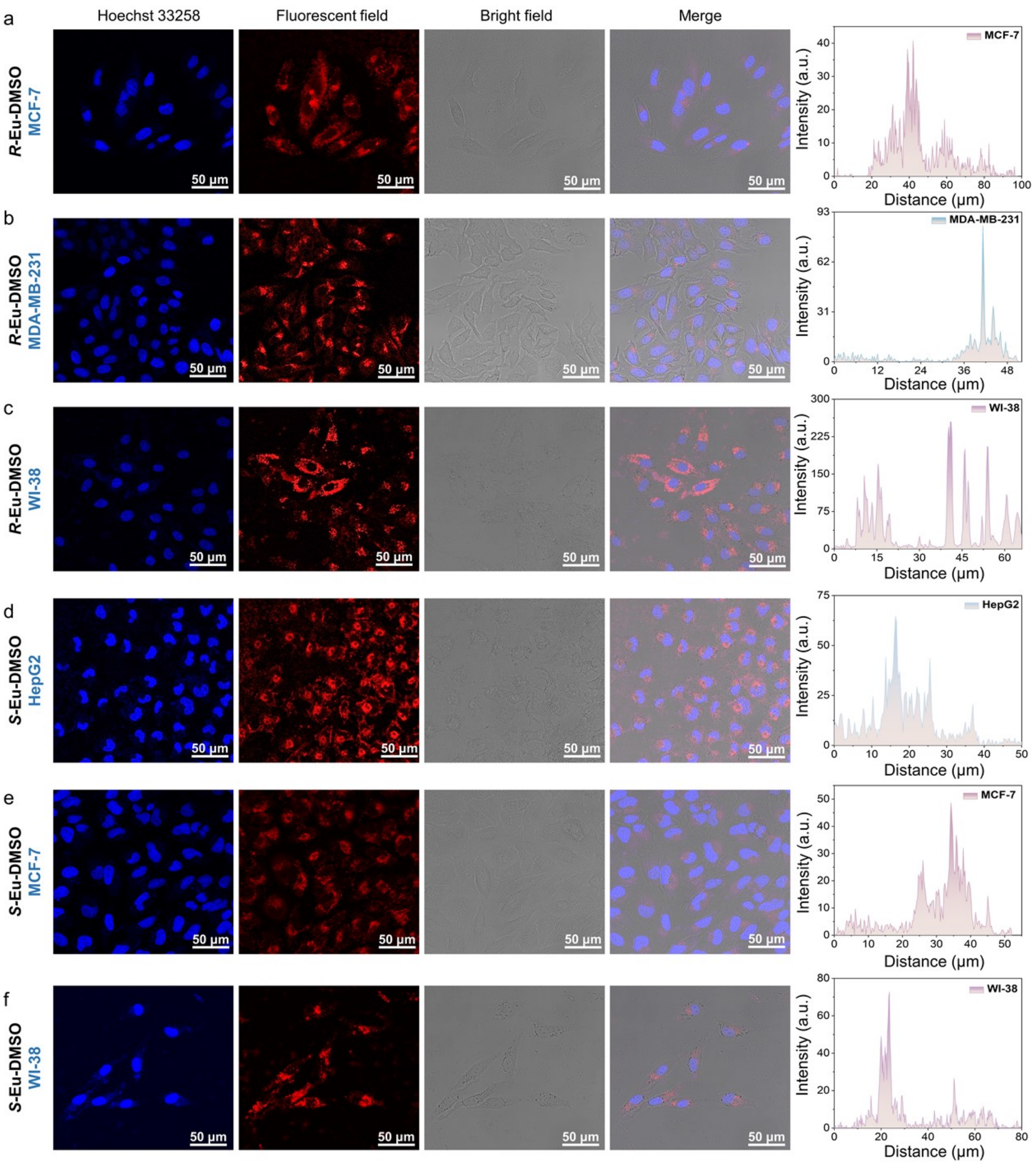

Figure S20. CLSM images and quantitative analysis of fluorescence intensity of ***R*-Eu-DMSO** after co-incubation with MCF-7 (a), MDA-MB-231 (b), and WI-38 (c); CLSM images and quantitative analysis of fluorescence intensity of ***S*-Eu-DMSO** after co-incubation with HepG2 (d), MCF-7 (e), and WI-38 (f).

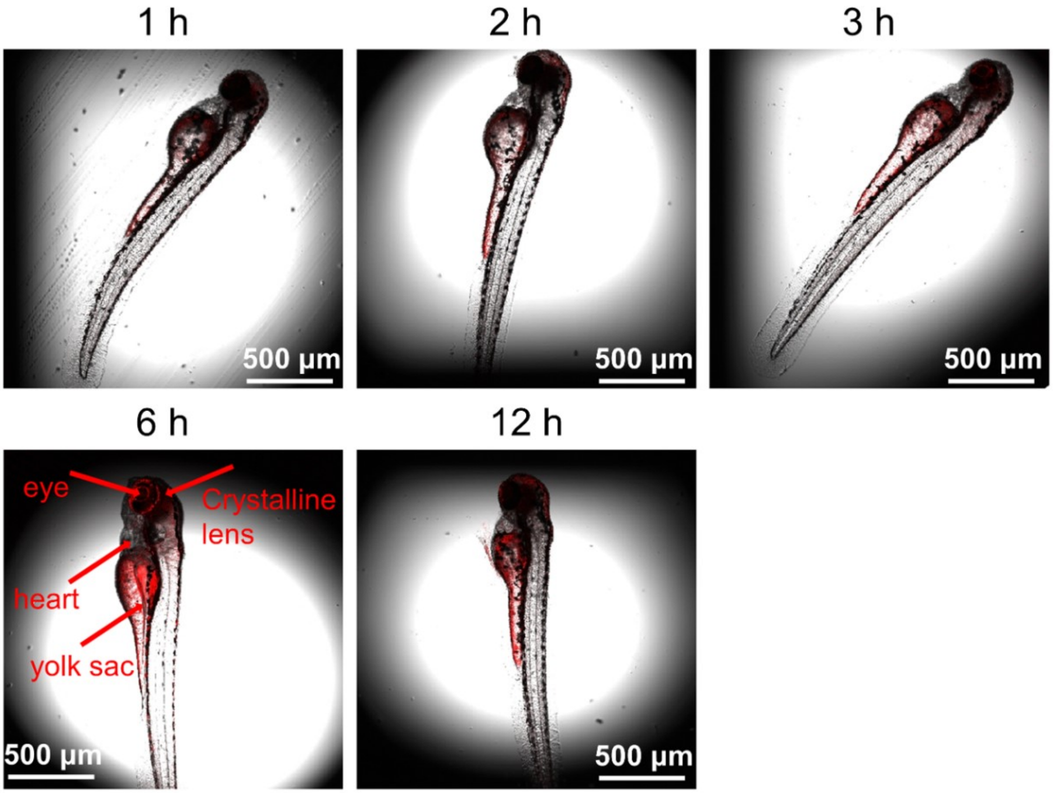

Figure S21. CLSM images of zebrafish co-incubated with ***R*-Eu-DMSO** for 1, 2, 3, 6, and 12 h, respectively.

Table S3. Selected bond lengths (Å) and angles (°) of ***R*****-Eu.**

**Bond lengths (Å)**			
Eu01-O3	2.512(4)	Eu02-O16	2.503(4)
Eu01-O7	2.493(4)	Eu02-O15	2.520(4)
Eu01-N7	2.613(4)	Eu02-O18	2.488(4)
Eu01-N6	2.527(5)	Eu02-N12	2.600(4)
Eu01-N4	2.551(5)	Eu02-O10	2.483(4)
Eu01-O9	2.542(4)	Eu02-O12	2.509(4)
Eu01-O6	2.513(4)	Eu02-O13	2.489(4)
Eu01-O2	2.502(4)	Eu02-N11	2.562(5)
Eu01-O01Z	2.519(4)	Eu02-N13	2.526(5)
Eu01-N5	2.559(4)	Eu02-N14	2.607(4)
**Bond angles (°)**			
O7-Eu01-O3	68.26(14)	O15-Eu02-O16	70.13(13)
N7-Eu01-O3	117.20(13)	O18-Eu02-O16	51.08(13)
N7-Eu01-O7	72.84(14)	O18-Eu02-O15	116.36(13)
N6-Eu01-O3	146.78(16)	N12-Eu02-O16	109.23(14)
N6-Eu01-O7	82.11(15)	N12-Eu02-O15	167.86(13)
N6-Eu01-N7	64.50(14)	N12-Eu02-O18	68.61(14)
N4-Eu01-O3	70.92(14)	O10-Eu02-O16	156.78(13)
N4-Eu01-O7	131.76(14)	O10-Eu02-O15	108.98(13)
N4-Eu01-N7	151.75(16)	O10-Eu02-O18	134.61(12)
N4-Eu01-N6	124.88(14)	O10-Eu02-N12	66.53(14)
O9-Eu01-O3	68.24(14)	O12-Eu02-O16	138.47(12)
O9-Eu01-O7	50.66(12)	O12-Eu02-O15	69.17(13)
O9-Eu01-N7	117.44(14)	O12-Eu02-O18	151.22(13)
O9-Eu01-N6	81.61(14)	O12-Eu02-N12	112.29(14)
O9-Eu01-N4	90.80(14)	O12-Eu02-O10	51.34(12)
O6-Eu01-O3	141.08(14)	O13-Eu02-O16	92.31(14)
O6-Eu01-O7	149.61(13)	O13-Eu02-O15	51.13(12)
O6-Eu01-N7	82.67(14)	O13-Eu02-O18	138.72(14)
O6-Eu01-N6	71.08(15)	O13-Eu02-N12	117.43(13)
O6-Eu01-N4	77.08(14)	O13-Eu02-O10	71.61(14)
O6-Eu01-O9	134.67(12)	O13-Eu02-O12	68.19(14)
O2-Eu01-O3	51.03(12)	N11-Eu02-O16	74.38(15)
O2-Eu01-O7	69.93(14)	N11-Eu02-O15	106.30(14)
O2-Eu01-N7	70.30(13)	N11-Eu02-O18	81.62(14)
O2-Eu01-N6	132.07(13)	N11-Eu02-N12	62.60(15)
O2-Eu01-N4	102.51(14)	N11-Eu02-O10	84.03(15)
O2-Eu01-O9	106.67(13)	N11-Eu02-O12	125.40(14)
O2-Eu01-O6	118.55(13)	N11-Eu02-O13	68.86(14)
O01Z-Eu01-O3	97.64(14)	N13-Eu02-O16	122.64(15)
O01Z-Eu01-O7	136.50(11)	N13-Eu02-O15	126.83(13)
O01Z-Eu01-N7	78.95(14)	N13-Eu02-O18	77.57(15)

O01Z-Eu01-N6	114.53(14)	N13-Eu02-N12	64.26(15)
O01Z-Eu01-N4	73.03(14)	N13-Eu02-O10	77.32(15)
O01Z-Eu01-O9	161.67(12)	N13-Eu02-O12	77.55(15)
O01Z-Eu01-O6	51.10(12)	N13-Eu02-O13	143.44(16)
O01Z-Eu01-O2	69.71(13)	N13-Eu02-N11	126.81(15)
N5-Eu01-O3	110.42(13)	N14-Eu02-O16	80.58(15)
N5-Eu01-O7	108.92(14)	N14-Eu02-O15	69.20(13)
N5-Eu01-N7	128.12(15)	N14-Eu02-O18	78.50(14)
N5-Eu01-N6	64.54(14)	N14-Eu02-N12	122.91(14)
N5-Eu01-N4	63.52(15)	N14-Eu02-O10	121.52(15)
N5-Eu01-O9	63.13(14)	N14-Eu02-O12	77.72(15)
N5-Eu01-O6	72.52(14)	N14-Eu02-O13	118.14(13)
N5-Eu01-O2	161.11(13)	N14-Eu02-N11	154.39(17)
N5-Eu01-O01Z	114.53(14)	N14-Eu02-N13	63.91(15)

Table S4. Selected bond lengths (Å) and angles (°) of ***S*-Eu.**

**Bond lengths (Å)**			
Eu1-N11	2.571(7)	Eu2-O9	2.534(7)
Eu1-O15	2.490(7)	Eu2-O2	2.497(6)
Eu1-N8	2.604(7)	Eu2-O8	2.494(6)
Eu1-N9	2.528(7)	Eu2-O5	2.521(7)
Eu1-O12	2.479(6)	Eu2-O3	2.523(6)
Eu1-O17	2.500(7)	Eu2-N2	2.561(6)
Eu1-O14	2.500(7)	Eu2-O6	2.507(7)
Eu1-N10	2.608(7)	Eu2-N4	2.603(7)
Eu1-O11	2.500(6)	Eu2-N3	2.525(7)
Eu1-O18	2.479(6)	Eu2-N1	2.533(6)
**Bond angles (°)**			
O15-Eu1-N11	81.8(2)	O2-Eu2-O9	106.7(2)
N8-Eu1-N11	154.7(2)	O8-Eu2-O9	51.50(19)
N8-Eu1-O15	78.7(2)	O8-Eu2-O2	69.5(2)
N9-Eu1-N11	126.6(2)	O5-Eu2-O9	161.89(19)
N9-Eu1-O15	77.4(2)	O5-Eu2-O2	69.6(2)
N9-Eu1-N8	64.2(2)	O5-Eu2-O8	135.64(18)
O12-Eu1-N11	69.2(2)	O3-Eu2-O9	67.9(2)
O12-Eu1-O15	139.2(2)	O3-Eu2-O2	50.8(2)
O12-Eu1-N8	117.6(2)	O3-Eu2-O8	67.8(2)
O12-Eu1-N9	143.2(3)	O3-Eu2-O5	98.2(2)
O17-Eu1-N11	125.6(2)	N2-Eu2-O9	63.1(2)
O17-Eu1-O15	150.6(2)	N2-Eu2-O2	160.9(2)
O17-Eu1-N8	77.3(2)	N2-Eu2-O8	109.6(2)
O17-Eu1-N9	77.1(2)	N2-Eu2-O5	114.6(2)
O17-Eu1-O12	68.4(2)	N2-Eu2-O3	110.6(2)
O14-Eu1-N11	73.9(2)	O6-Eu2-O9	134.72(19)

O14-Eu1-O15	51.0(2)	O6-Eu2-O2	118.4(2)
O14-Eu1-N8	81.3(2)	O6-Eu2-O8	149.8(2)
O14-Eu1-N9	122.8(2)	O6-Eu2-O5	50.78(19)
O14-Eu1-O12	92.4(2)	O6-Eu2-O3	141.1(2)
O14-Eu1-O17	139.43(19)	O6-Eu2-N2	72.5(2)
N10-Eu1-N11	62.6(2)	N4-Eu2-O9	117.8(2)
N10-Eu1-O15	68.5(2)	N4-Eu2-O2	70.9(2)
N10-Eu1-N8	123.1(2)	N4-Eu2-O8	72.7(2)
N10-Eu1-N9	64.1(2)	N4-Eu2-O5	78.4(2)
N10-Eu1-O12	117.7(2)	N4-Eu2-O3	117.4(2)
N10-Eu1-O17	112.0(2)	N4-Eu2-N2	127.7(2)
N10-Eu1-O14	108.6(2)	N4-Eu2-O6	82.5(2)
O11-Eu1-N11	106.9(2)	N3-Eu2-O9	82.0(2)
O11-Eu1-O15	116.6(2)	N3-Eu2-O2	132.4(2)
O11-Eu1-N8	68.6(2)	N3-Eu2-O8	82.5(2)
O11-Eu1-N9	126.5(2)	N3-Eu2-O5	114.0(2)
O11-Eu1-O12	51.2(2)	N3-Eu2-O3	146.6(2)
O11-Eu1-O17	69.2(2)	N3-Eu2-N2	64.5(2)
O11-Eu1-O14	71.0(2)	N3-Eu2-O6	71.2(2)
O11-Eu1-N10	168.3(2)	N3-Eu2-N4	64.2(2)
O18-Eu1-N11	83.6(2)	N1-Eu2-O9	90.8(2)
O18-Eu1-O15	134.14(19)	N1-Eu2-O2	101.8(2)
O18-Eu1-N8	121.7(2)	N1-Eu2-O8	132.1(2)
O18-Eu1-N9	77.2(2)	N1-Eu2-O5	73.1(2)
O18-Eu1-O12	71.7(2)	N1-Eu2-O3	71.1(2)
O18-Eu1-O17	51.65(18)	N1-Eu2-N2	64.0(2)
O18-Eu1-O14	156.1(2)	N1-Eu2-O6	76.9(2)
O18-Eu1-N10	66.2(2)	N1-Eu2-N4	151.3(2)
O18-Eu1-O11	109.2(2)	N1-Eu2-N3	125.2(2)

Table S5. *SHAPE* analysis of the Eu(III) ion in ***R*-Eu**.

Label	Shape	Symmetry	Distortion(°)
DP-10	D_{10h}	Decagon	35.576
EPY-10	C_{9v}	Enneagonal pyramid	24.044
OBPY-10	D_{8h}	Octagonal bipyramid	15.687
PPR-10	D_{5h}	Pentagonal prism	10.856
PAPR-10	D_{5d}	Pentagonal antiprism	10.735
JBCCU-10	D_{4h}	Bicapped cube J15	9.033
JBCSAPR-10	D_{4d}	Bicapped square antiprism J17	4.202
JMBIC-10	C_{2v}	Metabidiminished icosahedron J62	6.988
JATDI-10	C_{3v}	Augmented tridiminished icosahedron J64	20.012
JSPC-10	C_{2v}	Sphenocorona J87	3.437

SDD-10	D_2	Staggered Dodecahedron (2:6:2)	4.836
TD-10	C_{2v}	Tetradecahedron (2:6:2)	4.386
HD-10	D_{4h}	Hexadecahedron (2:6:2) or (1:4:4:1)	6.140

Table S6. *SHAPE* analysis of the Eu(III) ion in ***S*-Eu**.

Label	Shape	Symmetry	Distortion(°)
DP-10	D_{10h}	Decagon	34.717
EPY-10	C_{9v}	Enneagonal pyramid	23.832
OBPY-10	D_{8h}	Octagonal bipyramid	16.575
PPR-10	D_{5h}	Pentagonal prism	10.235
PAPR-10	D_{5d}	Pentagonal antiprism	9.359
JBCCU-10	D_{4h}	Bicapped cube J15	9.805
JBCSAPR-10	D_{4d}	Bicapped square antiprism J17	5.414
JMBIC-10	C_{2v}	Metabidiminished icosahedron J62	7.669
JATDI-10	C_{3v}	Augmented tridiminished icosahedron J64	19.114
JSPC-10	C_{2v}	Sphenocorona J87	3.049
SDD-10	D_2	Staggered Dodecahedron (2:6:2)	3.970
TD-10	C_{2v}	Tetradecahedron (2:6:2)	4.315
HD-10	D_{4h}	Hexadecahedron (2:6:2) or (1:4:4:1)	6.711

References

1 À. Tubau, F. Zinna, L. Di Bari, M. Font-Bardía and R. Vicente, *Dalton Trans.*, 2023, **52**, 1122-1132.

2 Z. Chen, W. Xu, C. Hu, Z. Liu, S. Lin, S. Guo, Z. Chen, M. Huang, Z.-Q. Yu, W.-H. Zhu and Y. Wu, *Inorg. Chem. Front.*, 2025. DOI: 10.1039/d5qi01288k.

3 À. Tubau, F. Zinna, L. Di Bari, M. Font-Bardía and R. Vicente, *Dalt. Trans.*, 2024, **53**, 13566-13582.

4 W.-W. Qin, B. Yu, G.-H. Zhang, M.-J. Tang, H.-H. Zou, F.-P. Liang and Z.-H. Zhu, *Rare Met.*, 2025. DOI: 10.1007/s12598-025-03524-3.

5 Y. Tang, M. Jian, B. Tang, Z. Zhu, Z. Wang and Y. Liu, *Inorg. Chem. Front.*, 2024, **11**, 2039-2048.

6 M. Tsurui, Y. Kitagawa, S. Shoji, K. Fushimi and Y. Hasegawa, *Dalton Trans.*, 2023, **52**, 796-805.

7 W. Li, S. Yin, Z. Song, P. Yan, Y. Zhou, T. Gao and H. Li, *Inorg. Chem. Front.*,

2025, **12**, 1176-1186.

8 M. Leonzio, M. Bettinelli, L. Arrico, M. Monari, L. Di Bari and F. Piccinelli, *Inorg. Chem.*, 2018, **57**, 10257-10264.

9 Y. Zhou, H. Li, T. Zhu, T. Gao and P. Yan, *J. Am. Chem. Soc.*, 2019, **141**, 19634-19643.

10 K. M. Ayers, N. D. Schley and G. Ung, *Inorg. Chem.*, 2020, **59**, 7657-7665.

11 M. Deng, N. D. Schley and G. Ung, *Chem. Commun.*, 2020, **56**, 14813-14816.

12 T. Zhao, Y. Zhang, G. Wang, X. Wang, P. Feng and S. Zang, *Angew. Chem. Int. Ed.*, 2025, **64**, e202421426.

13 B.-A. N. Willis, D. Schnable, N. D. Schley and G. Ung, *J. Am. Chem. Soc.*, 2022, **144**, 22421-22425.

14 Y.-L. Li, H.-L. Wang, Z.-H. Zhu, Y.-F. Wang, F.-P. Liang and H.-H. Zou, *Nat. Commun.*, 2024, **15**, 2896.

15 M. Tsurui, R. Takizawa, Y. Kitagawa, M. Wang, M. Kobayashi, T. Taketsugu and Y. Hasegawa, *Angew. Chem. Int. Ed.*, 2024, **63**, e202405584.
